# Development of a glycoconjugate vaccine to prevent invasive *Salmonella* Typhimurium infections in sub-Saharan Africa

**DOI:** 10.1371/journal.pntd.0005493

**Published:** 2017-04-07

**Authors:** Scott M. Baliban, Mingjun Yang, Girish Ramachandran, Brittany Curtis, Surekha Shridhar, Rachel S. Laufer, Jin Y. Wang, John Van Druff, Ellen E. Higginson, Nicolas Hegerle, Kristen M. Varney, James E. Galen, Sharon M. Tennant, Andrew Lees, Alexander D. MacKerell, Myron M. Levine, Raphael Simon

**Affiliations:** 1 Center for Vaccine Development, Institute for Global Health, University of Maryland School of Medicine, Baltimore, MD, United States of America; 2 Department of Medicine, University of Maryland School of Medicine, Baltimore, MD, United States of America; 3 University of Maryland Computer-Aided Drug Design Center and Department of Pharmaceutical Sciences, School of Pharmacy, Baltimore, MD, United States of America; 4 Fina Biosolutions, Rockville, MD, United States of America; 5 Department of Biochemistry and Molecular Biology, University of Maryland School of Medicine, Baltimore, MD, United States of America; 6 Department of Pediatrics, University of Maryland School of Medicine, Baltimore, MD, United States of America; Institut Pasteur, FRANCE

## Abstract

Invasive infections associated with non-typhoidal *Salmonella* (NTS) serovars Enteritidis (SE), Typhimurium (STm) and monophasic variant 1,4,[5],12:i:- are a major health problem in infants and young children in sub-Saharan Africa, and currently, there are no approved human NTS vaccines. NTS O-polysaccharides and flagellin proteins are protective antigens in animal models of invasive NTS infection. Conjugates of SE core and O-polysaccharide (COPS) chemically linked to SE flagellin have enhanced the anti-COPS immune response and protected mice against fatal challenge with a Malian SE blood isolate. We report herein the development of a STm glycoconjugate vaccine comprised of STm COPS conjugated to the homologous serovar phase 1 flagellin protein (FliC) with assessment of the role of COPS O-acetyls for functional immunity. Sun-type COPS conjugates linked through the polysaccharide reducing end to FliC were more immunogenic and protective in mice challenged with a Malian STm blood isolate than multipoint lattice conjugates (>95% vaccine efficacy [VE] versus 30–43% VE). Immunization with de-O-acetylated STm-COPS conjugated to CRM_197_ provided significant but reduced protection against STm challenge compared to mice immunized with native STm-COPS:CRM_197_ (63–74% VE versus 100% VE). Although OPS O-acetyls were highly immunogenic, post-vaccination sera that contained various O-acetyl epitope-specific antibody profiles displayed similar *in vitro* bactericidal activity when equivalent titers of anti-COPS IgG were assayed. *In-silico* molecular modeling further indicated that STm OPS forms a single dominant conformation, irrespective of O-acetylation, in which O-acetyls extend outward and are highly solvent exposed. These preclinical results establish important quality attributes for an STm vaccine that could be co-formulated with an SE-COPS:FliC glycoconjugate as a bivalent NTS vaccine for use in sub-Saharan Africa.

## Author summary

In sub-Saharan Africa, invasive non-typhoidal *Salmonella* (NTS) infections with serovars Enteritidis (SE) and Typhimurium (STm) are widespread in children, where up to 30% of cases are fatal. There are several licensed typhoid vaccines but no NTS vaccines. We previously reported that conjugates of SE lipopolysaccharide-derived core and O-polysaccharide (COPS) and the homologous serovar flagellin protein were effective vaccines against fatal infection in mice with a Malian SE invasive clinical isolate. We document herein the development of a promising STm vaccine candidate consisting of STm COPS conjugated to STm phase 1 flagellin (FliC). STm-COPS:FliC glycoconjugates induced robust immune responses and protected mice against fatal challenge with a Malian STm blood isolate. We additionally found that while COPS O-acetyl groups were strongly immunogenic, antibodies against non-O-acetyl polysaccharide backbone epitopes could also mediate bactericidal activity. Computational modeling further indicated that STm COPS assumes a single dominant conformation in which the O-acetyl groups are highly solvent exposed and thus available targets for the immune system. These results pave the way toward clinical studies of a bivalent NTS vaccine formulation.

## Introduction

Non-typhoidal *Salmonella* (NTS) are important human pathogens worldwide where infection in healthy adults normally results in self-limiting gastroenteritis. They can also cause fulminant invasive disease (e.g., bacteremia, septicemia, meningitis), particularly in hosts with immunological immaturity, immunosenescence, or immunosuppression [1]. In sub-Saharan Africa, hospital-based surveillance of pediatric patients admitted with fever or suspect focal bacterial infections (e.g., meningitis) revealed invasive NTS (iNTS) to be the major pathogen in infants and toddlers following *Haemophilus influenzae* type b (Hib) and *Streptococcus pneumoniae* (prior to implementation of Hib and pneumococcal conjugate vaccines) [2, 3]. iNTS case fatality rates of 15–30% are typical and most isolates are resistant to multiple antibiotics [1, 3]. Microbiological analyses of sub-Saharan iNTS isolates revealed that serogroup B serovars *S*. Typhimurium (STm) and 1,4,[5],12:i:- (that share the same phase 1 flagella type), and *S*. Enteritidis (SE), a serogroup D *Salmonella*, comprise 80–90% of isolates. Genomic sequencing of African STm isolates revealed the emergence and spread of a dominant rare multi-locus sequence type (MLST) 313 (rather than MLST 19 prevalent elsewhere globally). Sequencing also demonstrated genomic DNA loss and many pseudogenes including homologs found in *S*. Typhi or *S*. Paratyphi A [4, 5].

The bulk of sub-Saharan Africa iNTS disease occurs in children ≤5 years of age [1–3, 6]. The putative protective role of antibodies against iNTS disease in young children is supported by the observation that a higher proportion of infant cases occur after the first six months of life; by which time, maternal antibodies have waned [3, 7]. Given the limited number of serovars associated with disease burden, a vaccine immunoprophylaxis strategy is epidemiologically feasible. The success of field trials with Vi capsular polysaccharide (CPS) vaccines in typhoid endemic areas has established the paradigm for subunit vaccines comprised of *Salmonella* surface molecules to protect against invasive *Salmonella* infections. The O-polysaccharide (OPS) of lipopolysaccharide (LPS) and the flagellin subunit protein of flagellar filaments (H antigen) constitute prominent *Salmonella* cell surface structures and bear serovar-specific epitopes. Serogroups A, B and D OPS have a common →2)-α-D-Man*p*-(1→4)-α-L-Rha*p*-(1→3)-α-D-Gal*p*-(1→ trisaccharide backbone motif (antigen O12) that can be variably (1→6) glucosylated at galactose (antigen O1). An immunodominant dideoxy hexose linked α-(1→3) to mannose distinguishes these serogroups and is an abequose (antigen O4) for serogroup B. Group B OPS can also undergo O-acetylation at abequose C2 (generating antigen O5) or C2/3 of rhamnose as a consequence of phage lysogeny [8–11]. Studies in animal models have established that *Salmonella* OPS is an important virulence factor and is a target of protective antibodies for defense against invasive infection [12–16]. Unconjugated *Salmonella* OPS molecules are poorly immunogenic. However, covalent linkage to proteins improves their immunogenicity and enables development of OPS-based vaccines [17]. We documented that conjugates of SE COPS linked to the homologous serovar phase 1 flagellin protein are immunogenic and protect mice against fatal infection with a Malian SE blood isolate, and that antibodies against NTS flagellin proteins have bactericidal activity [18, 19]. We report here the development of a glycoconjugate vaccine comprised of STm COPS linked to the phase 1 flagellin protein from the same serovar, with exploration of the role that COPS O-acetyls provide in protective immunity.

## Materials and methods

### Bacterial strains, medium, and growth

The strains used in this study are described (S1 Table). All strains were maintained in Hi-Soy (HS) bacteriological media (5 g/L sodium chloride, 10 g/L soytone [Teknova, CA], 5 g/L Hy-yest [Sigma Aldrich, MO]) at 37°C. Growth and preparation of bacteria for *in-vitro* analyses and *in-vivo* infection was conducted as described [18]. Growth media for all *guaBA* mutants were supplemented with guanine; kanamycin was additionally supplemented for CVD 1925 (pSEC10-*wzzB*) (S1 Table). STm CVD 1925 (with or without pSEC10-*wzzB*) (S1 Table) and CVD 1943 (S1 Table) were grown in fully chemically defined media (CDM) in a fermenter. STm D65 (S1 Table) was grown in shake flasks. Fermentation conditions were as follows: 50 mL of CDM supplemented with 0.004% guanine was inoculated with 3–5 colonies from an HS agar plate and grown for 12–18 h at 37°C in a shake flask with agitation at 80 rpm. This culture was then used to inoculate 500 mL of CDM supplemented with 0.004% guanine that was grown under equivalent conditions for 8–10 h. Four liters of CDM containing 0.025% guanine was then inoculated to an OD_600_ nm of 0.15 with the 500 mL shake flask and maintained in a Biostat A-plus fermenter (Sartorius, Germany) culture, for 18–24 h at 400 rpm, 5 LPM ambient air, with an adjustment to pH 7 using 28% ammonium hydroxide.

### Protein purification and characterization

STm phase 1 flagellin proteins (FliC) were purified as described from culture supernatants of CVD 1925 [20]. Recombinant CRM_197_ produced in *E*. *coli* was obtained from Fina Biosolutions (Rockville, MD). FliC and CRM_197_ were confirmed for integrity and removal of residual host cell protein by SDS-PAGE with Coomassie Brilliant Blue staining, and endotoxin levels by Limulus Amebocyte Lysate (LAL) assay using the Endosafe PTS system (Charles River Laboratories, MA).

### COPS purification

COPS was harvested directly from LPS in the cell biomass and conditioned growth media of fermentation (CVD 1925 (pSEC10-*wzzB*), CVD 1943) or shake flask (STm D65) cultures by reducing the culture pH to 3.5–3.7 with glacial acetic acid, and incubation at 100°C for 4 h in glass bottles submerged in a boiling water bath. Post-hydrolysis supernatants were separated from insoluble material by centrifugation at 10k x g / 4°C for 30 min using a GS3 Rotor in a Sorvall RC5 refrigerated centrifuge. The supernatant fraction was brought to 1 M NaCl and filtered by tangential flow microfiltration through a 0.2 μm hollow-fiber filter (GE, NJ) at 4.5 psi transmembrane pressure (TMP) passing the full volume through followed by flushing with an equivalent volume of 1 M NaCl. The 0.2 μm cleared 1 M NaCl permeate was then concentrated 10-fold on a 30 kDa Hydrosart TFF membrane (Sartorius, Germany) at 14 psi TMP and diafiltered against 35 diavolumes of 1 M NaCl, followed by 10 diavolumes of 50 mM Tris pH 7. The retentate fraction in 50 mM Tris pH 7 was then passed through 3 x 3 mL Sartobind NanoQ anion exchange membranes (Sartorius, Germany) linked in series using an AKTA Purifier (GE, NJ) at 10 mL/min in 50 mM Tris pH 7. The flow-through fraction was brought to 25% ammonium sulfate and incubated overnight at 4°C. Precipitated material was removed by centrifugation at 10k x g / 4°C for 30 min using a GS3 rotor in a Sorvall RC5 refrigerated centrifuge followed by filtration through a 0.2 μm Stericup vacuum filter unit (Millipore, MA). Filtrates were then concentrated 10-fold by TFF with a Slice 200 TFF device using a 10 kDa Hydrosart membrane (Sartorius, Germany) at 7.5 psi TMP, and diafiltered against 10 diavolumes of de-ionized water. TFF retentates were lyophilized and stored at -20°C until use.

### COPS de-O-acetylation

For assessment of residual polysaccharide O-acetyls after exposure to different pH levels, 1925wzzB-COPS was incubated in 50 mM HEPES pH 7, 50 mM HEPES pH 8, 50 mM sodium borate pH 9, or 50 mM sodium borate pH 10 for 2 days at room temperature (RT). Complete de-O-acetylation was accomplished by incubation at pH 12 for 3 h at 37°C with pH adjustment and maintenance with 0.1M sodium hydroxide. Preparation of de-O-acetylated (dOAc) 1925wzzB-COPS for use as antigen in ELISA and vaccine preparation was accomplished by incubation in 50 mM sodium borate pH 10 for 2 days at RT.

### Carbohydrate analytical assays

High performance liquid size-exclusion chromatography (HPLC-SEC) analyses were performed with a Biosep SEC4000 column (Phenomenex, CA) on an Alliance 2795 (Waters, MA) run at 1 mL/minute with PBS pH 7.4. Absorbance at 280 nm and 252 nm were monitored with a 2487 dual-UV detector (Waters, MA) and refractive index with a 2414 refractive index detector (Waters, MA). Monosaccharide composition analyses were accomplished by depolymerization of purified polysaccharides in 1 M trifluoroacetic acid for 4 h at 100°C, followed by lyophilization, reconstitution in deionized water and filtration through a 0.2 μm syringe filter. Depolymerized samples were analyzed by high performance anion-exchange chromatography coupled with pulsed amperometric detection (HPAEC-PAD) using a CarboPac PA10 column run on a Dionex ICS4000 (Thermo Scientific, MA) at 0.010 mL/minute in 18 mM KOH and were compared to commercially available purified monosaccharide standards (Sigma Aldrich, MO) prepared under similar conditions. Analyses for O-acetylation were conducted by the method of Hestrin as described, with acetylcholine chloride standards (Sigma Aldrich, MO) [21]. Protein levels were assessed by bicinchoninic acid assay (Thermo-Pierce, MA) per the manufacturer’s instructions using purified bovine serum albumin (Sigma Aldrich, MO) as standards. Endotoxin levels were measured (LAL assay), and removal of nucleic acid was confirmed by absorbance at 260 nm. Size exclusion chromatography with multi angle light scattering (SEC-MALS) to determine the absolute molecular weight for purified COPS was performed using an Agilent 1100 HPLC system with an 8-angle Heleos detector and a Optilab T-rEX refractive index detector (Wyatt Technologies, CA). Fractionation was performed using TSKgel G4000 and 5000PWxl (Tosoh Biosciences, OH) in series with PBS + 0.02% sodium azide as the buffer at a flow rate of 0.5 mL/min. Analysis was performed using Astra 6.2 software (Wyatt Technologies, CA). The differential refractive index (dn/dC) necessary for the calculations was experimentally determined from purified, lyophilized COPS solubilized in the equilibration buffer, according to the protocol provided by the manufacturer. Resorcinol assays for carbohydrate concentration were conducted as described [18].

### Nuclear magnetic resonance (NMR)

Polysaccharides for NMR analyses were prepared by lyophilization and reconstitution in D_2_O. NMR spectra were recorded at 25°C on an 800 MHz (800.27 MHz for protons) Bruker Avance-series NMR spectrometer equipped with four frequency channels and a 5 mm triple-resonance z-axis gradient cryogenic probehead. A one-second relaxation delay was used, and quadrature detection in the indirect dimensions was obtained with states-TPPI phase cycling; initial delays in the indirect dimensions were set to give zero- and first-order phase corrections of 90° and –180°, respectively. Data were processed using the processing program nmrPipe on Mac OS X workstations. The ^1^H, ^13^C HSQC experiment was collected to monitor changes in the ^13^C and ^1^H resonances for O-acetylated and de-O-acetylated polysaccharides.

### LPS isolation and visualization by ProQ staining

Overnight bacterial cultures were adjusted to an OD_600_ of 1.0 and then 2 mL of culture was centrifuged at maximum speed for 2 min at 4°C. The supernatant was removed and the pellet resuspended in 100 μL lysis buffer (0.1 M Tris-HCl, pH 6.8, 2% SDS, 10% Glycerol, 4% 2-mercaptoethanol). The sample was boiled at 95–100°C for 10 min to lyse the cells. Proteins were digested by adding 25 μg Proteinase K. The sample was incubated at 60°C for 1 h. The sample was boiled for 10 min and then allowed to cool on ice. 20 μl of the sample was electrophoresed on 4–15% Mini Protean TGX stain-free gels (BioRad Laboratories, CA) with the CandyCane Glycoprotein ladder (Life Technologies, CA). LPS was stained using Pro-Q Emerald 300 LPS Gel Stain (Life Technologies, CA) as per the manufacturer’s instructions.

### Western blot analysis of lipopolysaccharide

Crude LPS extracts were made from several Malian isolates of STm (D65, A13, D23580, P142, Q65 and S42) (S1 Table) as well as SE R11 (S1 Table). Overnight bacterial cultures were normalized to an OD_600_ of 0.2, from which a 1 mL aliquot was centrifuged at 4°C for 10 min at 13,200 rpm. Pellets were resuspended in 100 μL of lysis buffer, vortexed vigorously and heated at 100°C for 10 min. Samples were cooled on ice followed by the addition of 25 μg of proteinase K. Samples were then incubated at 60°C for 1 h followed by 100°C for 10 min. LPS preparations were diluted with 100 μL of 2 x Laemmli sample buffer (Bio-Rad, Hercules, CA). Two volumes (10 μL and 2 μL) from each LPS sample were separated by electrophoresis on neutral pH, 1.5 mm, 4–12% Bis-Tris gels (Life Technologies, CA). The gels were wet transferred overnight at 4°C to methanol-activated polyvinylidene difluoride (PVDF) membranes and subsequently blocked with PBS + 0.05% Tween-20 pH 7.4 (PBST) + 10% Omniblok (AmericanBio, MA). To detect LPS, the membranes were then incubated with monoclonal IgA to STm O5 [clone Sal4, kind gift from Dr. Nicholas Mantis, Wadsworth Institute, NY] (1:1,000) or monoclonal IgG to *Salmonella* core polysaccharide (sc-52219, Santa Cruz Biotechnology, CA) (1:50) diluted in PBST + 10% Omniblok and incubated for 1 h at room temperature. Membranes were washed with PBST and incubated with either CruzFluor (CFL)-488-labeled anti-mouse IgA (for O5; 1:100) or CFL-647-labeled anti-mouse IgG (for core; 1:100) diluted in PBST + 10% Omniblok and incubated for 1 h at room temperature. All CFL-labeled antibodies were purchased from Santa Cruz Biotechnology, CA. Membranes were again washed and visualized using the Chemi-Doc MP system (Bio-Rad, CA).

### Preparation of 1925wzzB-COPS conjugates with FliC or CRM_197_

#### Lattice-type: Multipoint lattice formation with cyanylation chemistry

FliC monomer proteins at 5 mg/mL in 100 mM MES / 0.1% polysorbate 20, pH 6.5 were brought to 0.5 M adipic acid dihydrazide (ADH) and 5 mg/mL *N*-(3-Dimethylaminopropyl)-*N*′-ethylcarbodiimide. The reaction was incubated 12–16 h at 4°C at which point the labeled flagellin proteins were concentrated to 15 mg/mL and diafiltered against 10 diavolumes of 50 mM borate / 300 mM NaCl / 0.1% polysorbate 20, pH 9 with 30 kDa MicroKros hollow-fiber TFF filters (Spectrum Laboratories, CA). 1925wzzB-COPS was brought to 10 mg/mL and precooled by incubation on ice. Activation at random polysaccharide hydroxyls was achieved by addition of 100 mg/mL 1-cyano-4-dimethylaminopyridinium tetrafluoroborate (CDAP) in acetonitrile at a ratio of 0.5 mg CDAP per mg polysaccharide. The pH was raised to pH 9 using dimethylaminopyridine as the base. The reaction was incubated for 5 min on ice, at which point the activated polysaccharide was added to an equal amount of ADH-derivatized flagellin, and mixed by tumbling rotation for 2 h at room temperature and then 18 h at 4°C. The reaction was then quenched with 200 mM glycine.

#### Sun type: End-linkage at the 3-deoxy-D-*manno*-oct-2-ulosonic acid (KDO) terminus by aminooxy-thioether chemistry

Conjugation at the KDO carbonyl group present at the polysaccharide reducing end was accomplished with aminooxy chemistry as described [18, 22]. Briefly, 1925wzzB-COPS was suspended to 10 mg/mL in 100 mM sodium acetate pH 5 and brought to 5 mg/mL O-(3-mercaptopropyl)-hydroxylamine (Fina Biosolutions, MD). The reaction was incubated for 12–18 h at room temperature, at which point the labeled COPS was brought to 100 mM DTT, purified by desalting through Sephadex G25 in water, and lyophilized. FliC monomers or CRM_197_ were suspended to 10 mg/mL in 10 mM sodium phosphate pH 7.4 and derivatized with maleimide at protein amino groups directly prior to conjugation by reaction with a 30x molar excess of N-γ-maleimidobutyryl-oxysuccinimide ester (GMBS, Molecular BioSciences, CO) for 1 h at room temperature. Labeled proteins were then concentrated to 15 mg/mL with 30 kDa MicroKros hollow-fiber TFF filters (Spectrum Laboratories, CA) and purified by diafiltration against 10 diavolumes of 10 mM sodium phosphate / 5 mM EDTA, pH 6.8. Thiol-aminooxy labeled polysaccharide reconstituted to 20 mg/mL was added to the purified protein at a ratio of 3 mg polysaccharide per mg protein. The mixture was brought to 1x PBS, adjusted to pH 7.4 with 0.1 M NaOH, and incubated for 12 h at 4°C, at which point the reaction was quenched with 0.1 mM 2-mercaptoethanol.

#### Purification and characterization of COPS conjugates

Conjugates were separated from unreacted components by fractionation through a Superdex 200 10 x 300 GL size exclusion column with an AKTA Explorer (GE, NJ) run at 0.5 mL/min in PBS pH 7.4 with monitoring for absorbance at 280 nm and 260 nm. Individual fractions were assessed by HPLC-SEC and resorcinol assay as detailed. Selected fractions containing conjugates were pooled and filtered through 0.2 μm for use in animal immunization experiments.

### Immunization, sera collection and challenge

Eight to 10 week old female CD1 mice (Charles River Laboratories, MA) were injected intramuscularly (IM) in the right gastrocnemius at 0, 28 and 56 days with either sterile PBS (pH 7.4), 2.5 μg of STm FliC, or 2.5 μg polysaccharide conjugated to either STm FliC or CRM_197_. Sera were obtained before vaccination and three weeks after the final immunization. Four weeks after the final immunization (day 84), immunized mice were challenged intraperitoneally (IP) with STm D65 (LD_50_ = ~2 x 10^4^ colony forming units [CFU]) and monitored daily for 14 days after challenge while recording overall health, weight loss, and mortality. Mice that reached a moribund state (lethargy, non-responsiveness, dehydration, piloerection, and/or 48 h of sustained ≥20% weight loss) were euthanized and recorded as dead. Vaccine efficacy (VE) was calculated as [(proportional mortality in controls)-(proportional mortality in vaccine group)]/(proportional mortality in controls). Sera from mice immunized with CVD 1931 have been previously described [23]. Group sizes for challenge experiments were determined by the minimal number of mice required to provide ≥90% power to detect a significant difference for mortality rates in controls and vaccinees of ≥90% and ≤30% respectively (one-sided Fisher’s exact test, α = 0.025). Immunization, protection studies, and data analysis were done by two investigators blinded to the group allocations.

### Ethics statement

All animal studies were performed in facilities that are accredited by the Association for Assessment and Accreditation of Laboratory Animal Care and were in compliance with guidelines for animal care established by the US Department of Agriculture Animal Welfare Act, US Public Health Service policies, and US federal law. All animal experiments were in compliance with study protocols (0715008 and 0812010) approved by the University of Maryland School of Medicine Institutional Animal Care and Use Committee. Bacterial clinical isolates data had been de-identified and were analyzed anonymously.

### ELISA analyses for polysaccharide antigenicity analyses and titration of serum antibodies

Titration of serum IgG from STm vaccine-immunized mice, monoclonal antibodies (mAbs) (anti-*S*. Typhimurium O4 IgG [SC5223, Santa Cruz Biotechnology, CA]) and anti-*S*. Typhimurium O5 IgA (Sal4), or polyclonal anti-SE COPS sera (described previously [18]) was accomplished using an enzyme-linked immunosorbent assay (ELISA). Briefly, 96-well, medium-binding, microtiter plates (Greiner Bio-One, NC) were coated with either COPS antigens (1925wzzB-COPS, dOAc-1925wzzB-COPS, or SE COPS) or COPS conjugates (STm-COPS^KDO^:CRM_197_, or dOAc-STm-COPS^KDO^:CRM_197_) at a concentration of 5 μg polysaccharide/mL and incubated overnight at 4°C. Plates were washed with PBST and blocked with PBS + 10% Omniblok non-fat, dry milk for 2 h at 37°C. Serum samples and monoclonal antibodies were serially diluted in PBST + 10% Omniblok, transferred to blocked ELISA plates, and incubated for 1 h at 37°C. Plates were washed, and incubated for 1 h at 37°C with horseradish peroxidase (HRP)-labeled anti-mouse IgG (for O4 and mouse serum; 1:1,000) (KPL, MD) or HRP-labeled anti-mouse IgA (for O5; 1:500) (Southern Biotech, AL). After washing, substrate (3,3’,5,5’-tetramethylbenzidine, KPL, MD) was added, and the plates were incubated on a rocker at ambient temperature for 15 min in darkness. The reaction was stopped with the addition of 1 M H_3_PO_4_, and the absorbance at 450 nm was recorded using an Ascent microplate reader (Thermo Scientific, MA). Endpoint titers, represented as ELISA units (EU) per mL, were defined and calculated as previously described [18]. Analyses were conducted with positive sera as plate controls with acceptance criteria of < 15% variance between plates. Proportional levels of epitope-specific antibody in sera were calculated by subtracting the serum IgG titers for various COPS antigens as follows: O1,12,core antibody levels were defined as the SE COPS titer; O4 antibody levels were calculated as (dOAc-1925wzzB-COPS titer)–(SE COPS titer); O-acetyl specific antibody levels were calculated by (1925wzzB-COPS titer)–(dOAc-1925wzzB-COPS titer). Any negative values were assigned a titer of 0. The relative levels of each antibody population were calculated as a percentage of the sum of all three calculated titers.

### Serum bactericidal antibody (SBA) assay

Assays were conducted as described [24]. Briefly, the assay was prepared by first combining 25 μl of baby rabbit complement (BRC) (Pel-Freez Biologicals, AR), 15 μl of saline, and 50 μl of antibody sample and incubating with 10 μl of diluted bacteria (100–350 CFU). Negative controls contained the respective bacteria and complement only. Individual mouse sera were heat-inactivated at 56°C for 20 min prior to use in the assay. Antibody samples were adjusted prior to addition such that each sample contained an equivalent number of total anti-1925wzzB-COPS IgG EU in the 100 μl assay volume. Viable bacteria were determined after plating on rich media agar.

### Opsonophagocytic antibody (OPA) killing assay

Assays were conducted as described [25]. HL-60 cells were purchased from the American Type Culture Collection and were maintained in 1x RPMI-1640 medium supplemented with 10% [v/v] heat-inactivated fetal bovine serum prior to use. Briefly, 10 μl of bacterial suspension (~700–1000 CFU) was added to 25 μl of antibody in each well for opsonization at 37°C for 15 min in a 5% CO_2_ incubator, at which point 25 μl of BRC and 4 x 10^5^ HL-60 cells in 40 μl of media were added to each well. The 96 well plate was incubated at 37°C (no CO_2_) with shaking agitation at 160 rpm for 45 mins. Antibody samples were adjusted prior to addition such that each sample contained an equivalent number of total anti-1925wzzB-COPS IgG EU in the 100 μl assay volume. Negative controls were performed with buffer in place of sera. Viable bacteria were determined after plating on rich media agar.

### Computational analyses

#### Hamiltonian replica exchange molecular dynamics simulation method

All simulations were performed with the program CHARMM [26] under the CHARMM36 additive force field for carbohydrates [27], except the simulation of the O-acetylated saccharide, which was carried out with CHARMM interfaced to OpenMM [28]. As part of the present study, empirical force field parameters for the O-acetyl groups were optimized as described below. Molecular models of polysaccharides were constructed to study the impact of acetylation and glucosylation on the conformational preferences of the antigen saccharides. These include the base polysaccharide with 3 tetrasaccharide repeat units, the O-acetylated saccharide at C2 position of both of the α-D-Abe*p* and α-L-Rha*p* residues, the saccharide with glucosylation at either the central repeat α-D-Gal*p*(7) (glucosylated saccharide 1) or terminal repeat α-D-Gal*p*(11) (glucosylated saccharide 2) via a 1→4 glycosidic linkage (GL) in the absence or presence of the O-acetylations. Each system was immersed in a cubic waterbox based on the CHARMM-TIP3P water model [29] with size of 55Å×55Å×55Å. In all simulations, the temperature was maintained at 298 K using the Hoover algorithm with a thermal piston mass of 1000 kcal/mol·ps^2^ and a constant pressure of 1 atm was realized using the Langevin piston algorithm with a collision frequency of 20 ps^-1^ and mass of 1630 amu [30]. Covalent bonds involving hydrogens were constrained with SHAKE, which allows an integration step of 2 fs in the molecular dynamics (MD) simulations. The non-bonded Lennard-Jones interactions were computed within a cutoff of 12 Å with a force switching function applied over the range from 10 to 12 Å. The electrostatic interactions were treated by the particle mesh Ewald method with a real space cutoff of 12 Å, a charge grid of 1 Å, and a 6-th order spline function for mesh interpolation. The Hamiltonian replica exchange with concurrent solute scaling and biasing potential (HREST-BP) method was adopted to obtain adequate conformational sampling of each solvated system [31]. Because the conformational propensity of the antigen saccharides is of interest, only the carbohydrate molecule in each system was treated as a solute in the HREST-BP simulations and subjected to concurrent potential biasing along every GL and temperature scaling of the intra-solute potential and solute-environment interactions. The biasing potential was based on 2-dimensional grid based correction maps (bpCMAP) along the two torsional angles ϕ(O_5_-C_1_-O_n_-C_n_)/Ψ(C_1_-O_n_-C_n_-C_n+1_) for each GL and was constructed using the corresponding disaccharide model as described in a previous study [31]. For each simulation, 6 replicas were used in the HREST-BP simulation and exchanges were attempted every 1000 MD steps according to the Metropolis criterion. The ground-state replica was simulated unperturbed at 298 K and the other 5 perturbed replicas were assigned a scaling temperature of 316 K, 335 K, 356 K, 377 K and 400 K, respectively. The distribution of scaling factors was derived following the scheme described in the HREST-BP paper for the biasing potentials [31]. S2 Table includes the simulation length for each system.

#### Glycosidic linkage-based clustering analysis (GL clustering)

Conformations of the saccharides were identified using clustering analysis performed on the basis of glycosidic linkages (GL), allowing for characterization of the conformational heterogeneity of each saccharide molecule [32]. The conformation of each GL can be represented with the ϕ and Ψ torsion angles. This allows for the 2D GL ϕ/Ψ surfaces to be partitioned into 4 quadrants corresponding to local minima (S1 Fig), with clustering based on index number identifiers of the quadrants (i.e. 1, 2, 3, 4). S3 Table shows the definition of the ϕ/Ψ values on the free energy surfaces corresponding to different quadrants. All 1→3 and 1→4 linkages were included in the clustering analysis; the 1→2 linkages were not used since only one minimum was sampled along the torsion angles ϕ/Ψ. From every snapshot of the simulation, the index number that denotes the state of each GL was obtained to define the overall conformation of the polysaccharide. Given the presence of eight 1→3 and 1→4 linkages, the specific combination of the eight index numbers for clustering defines that cluster. For example, 11111111 indicates that all linkages are located in the region of ϕϵ(-180°, -120°) or (0°, 180°]/Ψϵ(-180°, -120°) or (0°, 180°]. In this clustering approach, all GL conformations are based on a cluster occupying the same local free energy minima for all the linkages (referred to as GL clusters). We note that conformations in the same cluster can vary in terms of Cartesian coordinates; however, as they occupy the same minima, these different “Cartesian conformations” can readily interconvert without encountering any high free energy barriers. GL clusters, therefore, provide a high dimensional representation of the carbohydrate conformation.

#### 3D spatial distribution of the saccharide conformations

The spatial distribution, or range of conformational space as defined by Cartesian coordinates, can be represented by the 3D spatial volume of the sampled conformations. To compute the sampled spatial volumes, a 3D grid with a voxel size of 1Å0××1Å×1Å was firstly constructed around each saccharide. Then, for a given snapshot from the individual simulations, each voxel was assigned a value of 1 if it was occupied by a non-hydrogen atom of an aligned frame. The occupied volume refers to the total number of occupied voxels. This analysis was performed for all snapshots from the respective trajectories. Visualization of the sampled volume was performed by normalizing the voxel occupancies by all the snapshots. This allows for the volume sampled by selected portions of the saccharides, such as the O-acetyl groups as well as of the full saccharide, to be quantified and visualized.

#### Empirical force field parametrization of the O-acetyl group

Parameters of the O-acetyl group were derived following the parametrization philosophy for CHARMM36 additive force field of carbohydrates [27]. A quantum mechanical (QM) scan along the torsional angle C3-C2-O2-CA2 was carried out every 15° in acetylated α-D-Abequ*p* and α-L-Rha*p* at the C2 position (S2 Fig). Each conformation was firstly optimized at the RIMP2/6-31G* level in Q-CHEM [33] and then the single-point energy was computed at the MP2/cc-pVTZ level with PSI4 [34] to serve as the target data. The torsional parameters for the dihedral C3-C2-O2-CA2 in both molecular models were refitted against the QM conformational energies with the *lsfitpar* program for robust fitting of bonded parameters [35]. Empirical energy surfaces were computed by restraining the target dihedrals with a force constant of 9999 kcal/mol/rad^2^, with the remaining degrees of freedom minimized to a gradient of 10^−4^ using the steepest descent followed by the conjugate gradient minimizers (S3 Fig). The initial, unoptimized model with transferred parameters had a root mean square difference of 3.34 and 1.32 kcal/mol between empirical and QM energies for all conformations with the RMSD reduced to 2.31 and 0.92 kcal/mol in the final model for acetylated α-D-Abequ*p* and α-L-Rha*p*, respectively. The parameters and topologies for the O-acetylation patches of hexopyranose are included in the latest release of the CHARMM36 additive force field (http://mackerell.umaryland.edu/charmm_ff.shtml).

### Statistical analyses

All statistical analyses were performed using GraphPad Prism v6 (GraphPad Software, CA). No animals were excluded from analysis. For ELISA analyses, the majority of data did not meet the criteria for Gaussian distribution. Therefore, all comparisons between groups were conducted using non-parametric tests: either a two-tailed Mann-Whitney U test for unpaired samples or two-tailed Wilcoxon signed rank test for paired samples (α = 0.05 for both tests). No adjustment was made for multiple comparisons. Survival analysis after active immunization was assessed by the log-rank test. Comparisons of either SBA or OPA fitted curves were made using a nonlinear regression analysis (extra sum-of-squares F test, α = 0.05). *P* values of ≤ 0.05 were considered statistically significant.

## Results

### Polysaccharide characterization

**Fig 1:**
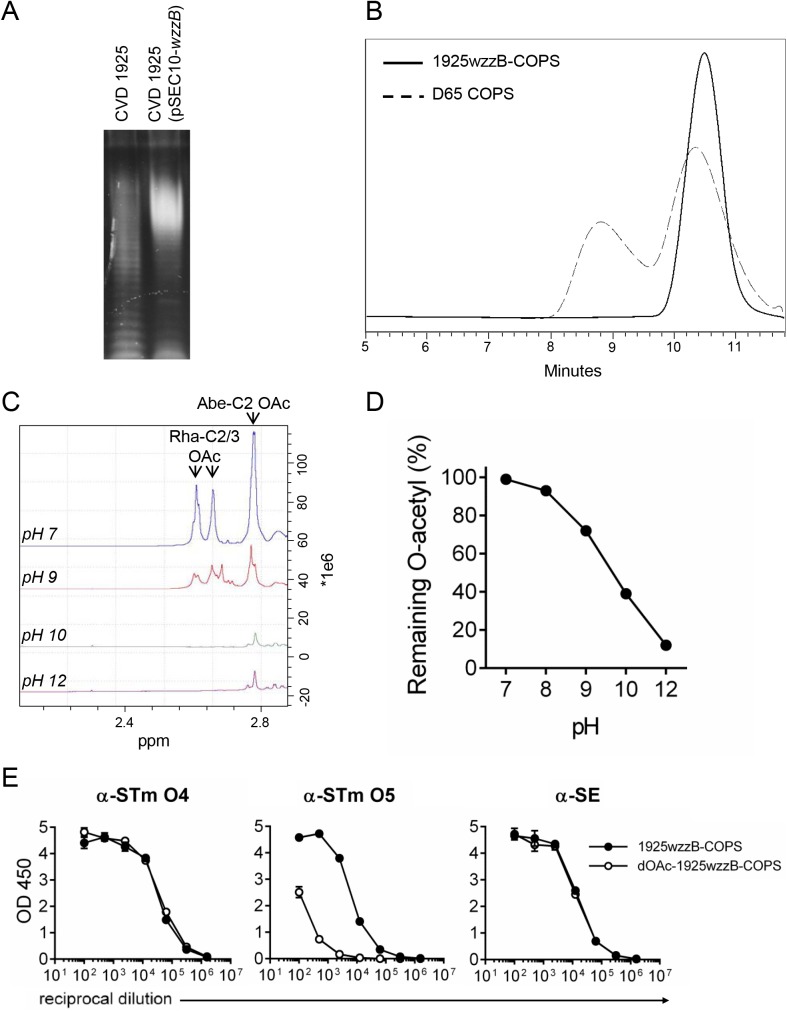
STm COPS size characterization and the effect of pH on rhamnose and abequose O-acetylation in 1925wzzB-COPS. (A) SDS-PAGE and Pro-Q staining for LPS from CVD 1925 (lane 1) and CVD 1925 (pSEC10-*wzzB*) (lane 2). (B) Chromatogram of purified D65 COPS (dashed line) and 1925wzzB-COPS (solid line) assessed by HPLC-SEC with detection by refractive index. (C) ^1^H NMR analysis of 1925wzzB-COPS after exposure to different pH levels. Peaks for rhamnose C2/C3 and abequose C2 O-acetyls are indicated. (D) Hestrin analysis of residual O-acetylation of 1925wzzB-COPS after incubation at different pH levels. (E) ELISA reactivity of 1925wzzB-COPS and dOAc-1925wzzB-COPS with monoclonal antibodies against O4 (α-STm O4) and O5 (α-STm-O5) or polyclonal sera recognizing O1,12 and core epitopes (α-SE). Error bars represent s.d. and were derived from technical replicates from one experiment.

**Fig 2:**
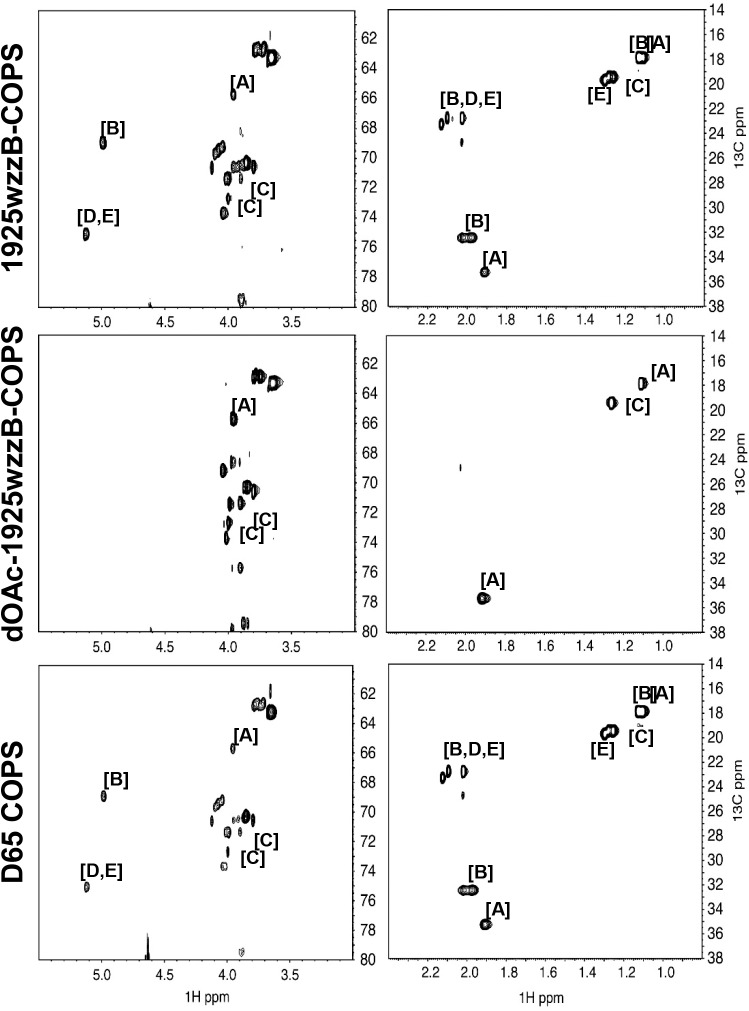
^1^H and ^13^C NMR analysis of native and de-O-acetylated 1925wzzB-COPS and native D65 COPS. ^1^H and ^13^C spectra obtained by 800 MHz NMR are shown for 1925wzzB-COPS, dOAc-1925wzzB-COPS, and D65 COPS. NMR shifts for O-acetylated and native monosaccharides within the COPS polymer are differentiated by letter: α-D-Abequ*p* [A], α-D-Abequ*p* + OAc at C2 [B], α-L-Rha*p* [C], α-L-Rha*p* + OAc at C2 [D], α-L-Rha*p* + OAc at C3 [E].

STm COPS for use as a vaccine antigen was purified from CVD 1925 (pSEC10-*wzzB*), a recombinant strain engineered to express long-chain OPS due to overexpression of *wzzB*, a member of the polysaccharide co-polymerase family (Fig 1A) [36, 37]. CVD 1925 (pSEC10-*wzzB*) COPS (1925wzzB-COPS) demonstrated a single, sharply-defined population determined to be ~19.8 kDa by SEC-MALS (Fig 1B). HPAEC-PAD analysis confirmed glucosylation at ~12% of OPS repeats based on the ratio with rhamnose (S4 Table). NMR analyses indicated variable O-acetylation at abequose C2 and C2/3 of rhamnose (Fig 2 and Table 1) [38]. 1925wzzB-COPS was recognized by monoclonal antibodies specific for the O4 and O5 antigens as well as polyclonal sera recognizing O1,12, and core epitopes (derived from mice immunized with an SE-COPS:FliC conjugate) (Fig 1E). A comparable pattern of glucosylation (S4 Table) and O-acetylation (Fig 2 and Table 1) was found for the OPS of STm strain D65, a previously described Malian ST313 blood isolate used herein for challenge studies [23]. D65 COPS demonstrated a bimodal size distribution with a population equivalent in size to 1925wzzB-COPS, as well as a higher molecular weight species (Fig 1B).

**Table 1:** ^1^H and ^13^C values for different *S*. Typhimurium COPS residues.

Residue		H1/C1	H2/C2	H3/C3	H4/C4	H5/C5	H6/C6	OAc methyl
Abe [A]	Reference^a^	5.09/102.1	4.02/64.8	1.98/34.5	3.87/69.7	4.12/68.2	1.18/16.9	
	Observed	5.08/104.0	3.96/65.7	1.91/35.3			1.11/17.8	
Abe-OAc [B]	Reference	5.27/99.4	5.1/68.1	2.12/31.6	3.9/69.6	4.19/68.6	1.23/16.9	2.13/21.8
	Observed	5.17/100.3	4.99/68.9	1.98/32.4			1.13/17.8	2.13/23.3
Rha [C]	Reference	5.09/102.9	4.11/71.7	3.99/70.5	3.59/83	3.96/69.4	1.35/18.5	
	Observed	5.00/103.9	4.00/72.7	3.89/71.4			1.26/19.5	
Rha-C2-OAc [D]	Reference	5.12/100.3	5.23/74.2	4.22/68.8	3.63/83	4.04/69.4	1.38/18.5	2.19/21.9
	Observed		5.13/75.1				1.29/19.4	2.10/22.8
Rha-C3-OAc [E]	Reference	5.10/102.9	4.23/69.7	5.21/74.2	3.85/79.5	4.05/69.4	1.41/18.8	2.23/22.2
	Observed	5.02/102.9		5.12/75.11			1.30/19.7	2.13/23.3

^a^ adapted from ref. [38].

Polysaccharide O-acetyl groups are stable at neutral pH but labile under alkaline conditions. To assess the site-specific susceptibility of O-acetyls to base treatment, residual O-acetylation in 1925wzzB-COPS was assessed after exposure to different pH conditions. O-acetylation was maintained at pH 7–8 but lost at approximately equivalent levels from abequose and rhamnose at ≥pH 9 (Figs 1C, 1D and 2 and Table 1). ELISA analysis confirmed marked O5 loss at pH 10, while maintaining O1,4,12 and core antigenicity (Fig 1E).

### Anti-COPS IgG in sera from mice immunized with live-attenuated STm

**Fig 3:**
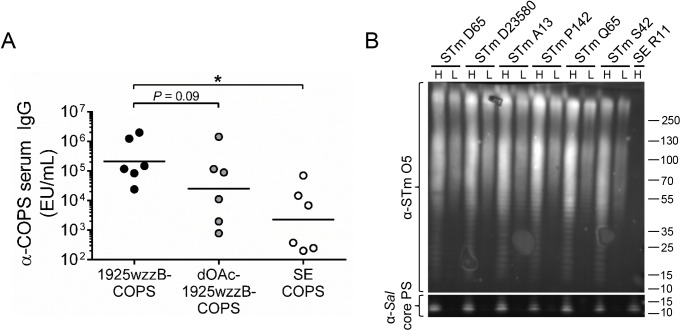
COPS epitope selectivity for sera from mice immunized with a live-attenuated STm vaccine. (A) Serum IgG titers for 1925wzzB-COPS (black), dOAc-1925wzzB-COPS (grey), and SE COPS (open) from mice immunized with CVD 1931 (*n* = 6). Each point represents an individual mouse. Solid bars indicate the GMT; comparisons between paired serological analyses were accomplished by two-tailed Wilcoxon signed-rank test, *P ≤ 0.05. (B) Western blot of crude LPS extracts from stationary-phase growth cultures of various *Salmonella* strains (detailed in S1 Table). Two volumes from each extract were separated: H, “high” 10 μL; L, “low” 2 μL. Abequose O-acetyl groups were detected with a STm O5-specific monoclonal antibody and normalized to reactivity with an anti-core polysaccharide monoclonal antibody [α-*Sal* core PS].

Characterization of the immune response to bacterial surface polysaccharides after exposure to the whole organism provides important information for development of subunit vaccines based on these antigens. Alkaline treatment of COPS is also effective for analyzing antibody responses to polysaccharide O-acetyls, as other attributes such as size and backbone structure remain unchanged [10, 39]. Accordingly, we measured antibody titers against native and pH-10, de-O-acetylated (dOAc-)1925wzzB-COPS as well as SE COPS in sera from mice immunized with CVD 1931, an attenuated vaccine strain derived from STm strain D65 that mediates robust protection against wild-type D65 challenge [23]. In these sera, anti-COPS IgG recognized primarily STm-specific O-epitopes (1925wzzB-COPS vs. SE COPS, *P* < 0.05), and IgG titers were generally higher for native compared to dOAc-1925wzzB-COPS (*P* = 0.09, Fig 3A). These data suggest that antibodies to O-acetylated epitopes are induced under conditions where protection is achieved. To confirm that COPS from strain D65 was representative of other circulating STm strains in Mali, we assessed LPS from isolates obtained in different years from l’Hôpital Gabriel Touŕe in Bamako, Mali. Western blots performed with an anti-O5 mAb revealed comparable LPS banding patterns and intensities for all STm strains analyzed, including Malawian STm D23580 (Fig 3B).

### Immunogenicity and protection from STm-COPS:FliC glycoconjugates synthesized with different chemistries

**Fig 4:**
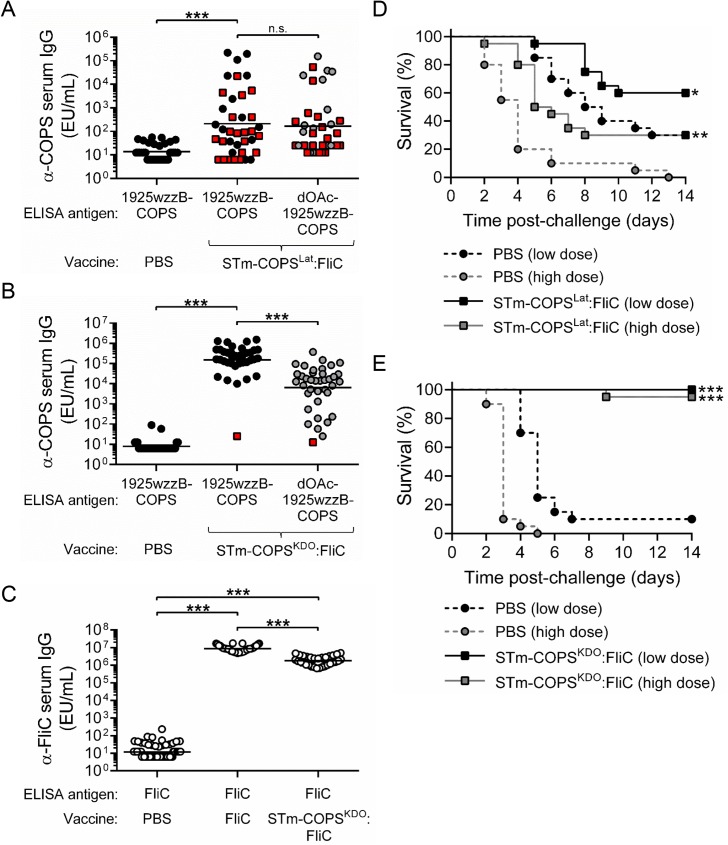
Immunogenicity and protection against STm infection in mice immunized with different COPS:FliC conjugates or unconjugated flagellin. (A,B,C) Serum IgG titers for 1925wzzB-COPS (black), dOAc-1925wzzB-COPS (grey) or STm FliC (open) from mice (*n* = 40/group) immunized with PBS or STm-COPS^Lat^:FliC (A), STm-COPS^KDO^:FliC (B,C),or STm FliC (C). Each point represents an individual mouse. Red squares indicate mice that succumbed to challenge. Solid bars indicate the GMT; comparisons between groups were accomplished by either a two-tailed Mann-Whitney U test (PBS vs. conjugate) or two-tailed Wilcoxon signed-rank test (paired conjugate sera analyses). (D,E) Kaplan-Meier survival curves of mice immunized with PBS (circles, dashed lines) or conjugates of STm-COPS^Lat^:FliC (D) or STm-COPS^KDO^:FliC (E) (squares, solid lines) after challenge (*n* = 20/group) with 1x10^5^ CFU (black) or 5x10^5^ CFU (grey) of STm D65. Survival curves were compared using log rank analysis. Adjustments for multiple comparisons were not made. *P ≤ 0.05, **P ≤ 0.001, ***P ≤ 0.0001, n.s. not-significant.

Conjugates of STm COPS and FliC were generated by different conjugation methods. We initially produced lattice-type conjugates (S4A Fig) by multipoint conjugation between random 1925wzzB-COPS hydroxyls and amino groups on ADH-derivatized FliC using CDAP (STm-COPS^Lat^:FliC) [40]. Maximal linkage between polysaccharide and protein by CDAP requires pH 9–10 conditions during conjugation [40]. Accordingly, while efficient formation of high molecular weight conjugates was observed (S4B Fig), we found marked loss of polysaccharide O-acetyls after conjugation (Table 2). Mice immunized with this conjugate generated higher geometric mean titers (GMTs) of anti-1925wzzB-COPS IgG compared to controls administered PBS; however, the GMTs were low, and no difference was seen when the sera were screened against dOAc-1925wzzB-COPS (Fig 4A). Challenge with 1x10^5^ or 5x10^5^ CFU of STm D65 produced 70% and 100% mortality, respectively, in PBS controls, whereas immunization with the STm-COPS^Lat^:FliC conjugate provided 43% and 30% protection against mortality following these challenge doses (Fig 4D).

**Table 2:** Biophysical and biochemical attributes of STm COPS conjugates used in this study.

Conjugate	Chemistry	Linker	PS:protein^a^ (final)	O-acetyl level^b^
STm-COPS^Lat^:FliC	CDAP	ADH	0.75	31%
STm-COPS^KDO^:FliC	Aminooxy-thioether	Thiol-aminooxy/GMBS	1.4	100%
STm-COPS^KDO^:CRM_197_	Aminooxy-thioether	Thiol-aminooxy/GMBS	2.2	100%
dOAc-STm-COPS^KDO^:CRM_197_	Aminooxy-thioether	Thiol-aminooxy/GMBS	2.4	11%

^a^ Weight:weight.

^b^ relative to 1925wzzB-COPS by Hestrin assay.

To produce a conjugate formulation that retained OPS O-acetyls, sun-type conjugates (S4A Fig) were generated by functionalization of the KDO carbonyl at the reducing end of 1925wzzB-COPS with an aminooxy thiol reagent. This yielded a free thiol that was then coupled to maleimide-derivatized protein lysines (STm-COPS^KDO^:FliC). This approach allowed the entire conjugation to ensue at neutral pH. Conjugates generated by this method maintained O-acetylation levels comparable with the native polysaccharide (Table 2). Mice immunized with STm-COPS^KDO^:FliC manifested robust anti-1925wzzB-COPS IgG titers with a GMT ~1,000-fold higher than was seen for the lattice conjugate (Fig 4B). Importantly, we found that the GMT of antibody to native 1925wzzB-COPS was ~10-fold higher than was seen for antibodies directed against dOAc-1925wzzB-COPS and thus was similar to the profile found for sera from CVD 1931-immunized mice (Fig 3A). Immunization with this conjugate also induced high anti-FliC titers in all mice comparable to titers achieved after immunization with unconjugated FliC (Fig 4C). Challenge with both low (1x10^5^ CFU) and high (5x10^5^ CFU) doses of STm D65 was sufficient to cause >90% mortality in unimmunized controls, with mice given the higher dose succumbing more rapidly (Fig 4E). Alternatively, mice immunized with STm-COPS^KDO^:FliC were protected against fatal infection at both of these challenge doses (95–100% VE).

### Immunogenicity and protective activity of end-linked conjugates synthesized with CRM_197_ and native or dOAc COPS

**Fig 5:**
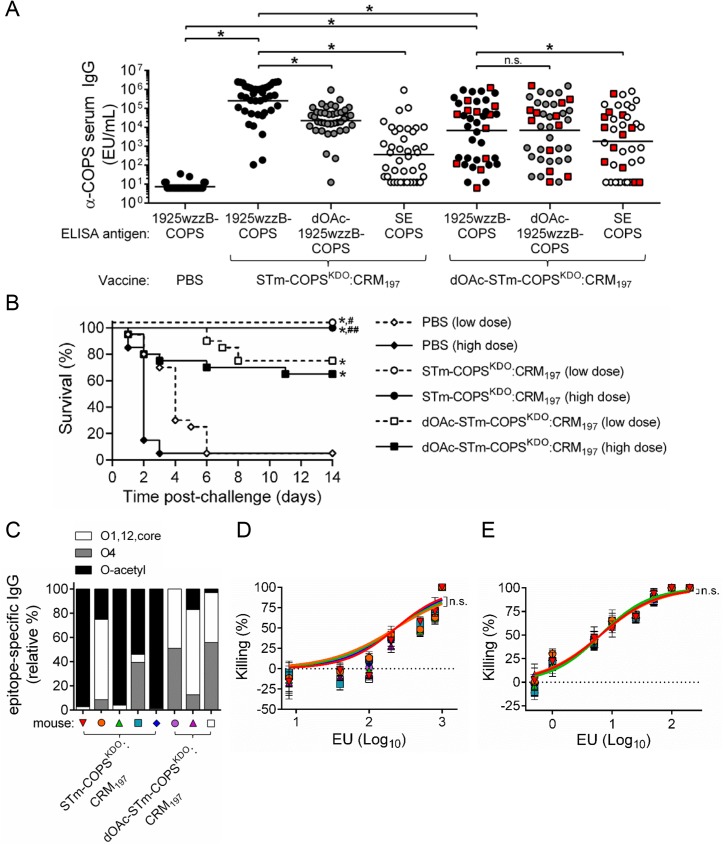
Immunogenicity, protective efficacy and functional analyses of vaccine-induced antibody for mice immunized with conjugates of native or dOAc-1925wzzB-COPS with CRM_197_. (A) Serum IgG titers for 1925wzzB-COPS (black), dOAc-1925wzzB-COPS (grey) or SE COPS (open) from mice (*n* = 40/group) immunized with PBS, STm-COPS^KDO^:CRM_197_ or dOAc-STm-COPS^KDO^:CRM_197_ as indicated. Each point represents an individual mouse. Red squares indicate mice that succumbed after challenge. Solid bars indicate the GMT; comparisons between groups were accomplished by either a two-tailed Mann-Whitney U test (PBS vs. conjugate, conjugate 1 vs. conjugate 2) or two-tailed Wilcoxon signed-rank test (paired serological analyses). (B) Kaplan-Meier survival curves of mice immunized with STm-COPS^KDO^:CRM_197_ (circle), dOAc-STm-COPS^KDO^:CRM_197_ (square) or PBS (diamond) after challenge (*n* = 20/group) with 1x10^6^ CFU (open symbol, dashed line) or 5x10^6^ CFU (closed symbol, solid line) of STm D65. Adjustments for multiple comparisons were not made. *P ≤ 0.0001 for indicated comparisons or for vaccinated mice relative to the respective PBS challenge group. ^#^*P* ≤ 0.05, ^##^*P* ≤ 0.005 for STm-COPS^KDO^:CRM_197_ vaccinated mice relative to the respective challenge dose group immunized with dOAc-STm-COPS^KDO^:CRM_197_. (C) Representative serum anti-COPS epitope profiles (identified by symbol) from mice immunized with either STm-COPS^KDO^:CRM_197_ or dOAc-STm-COPS^KDO^:CRM_197_. Selected sera were chosen to assess serum bactericidal antibodies (“SBA”, D) and opsonophagocytic antibodies (“OPA”, E) and were diluted such that each sample contained equivalent anti-1925wzzB-COPS IgG EU. Curves were fitted to each serial dilution of serum and were compared using nonlinear regression analysis. Dashed line indicates 0% killing. Results are representative of two independent assays, error bars represent s.d. and were derived from technical replicates; n.s., not significant.

To determine the functional relevance of O-acetyl groups in the context of equivalent conjugate architecture and the absence of other STm antigens, we assessed the immunogenicity and protection imparted by native and dOAc-1925wzzB-COPS thioether-linked sun-type conjugates with CRM_197_ ([dOAc-]STm-COPS^KDO^:CRM_197_). Antigenicity analyses of the conjugated COPS confirmed retention of the O4 antigen in both formulations but marked loss of O5 in the dOAc-1925wzzB-COPS conjugate (S5 Fig). Sera from mice immunized with STm-COPS^KDO^:CRM_197_ demonstrated high titers of anti-COPS IgG against the native polysaccharide (Fig 5A) that were similar to the GMT achieved by sun-type conjugates of native COPS with FliC (Fig 4B). The IgG GMT for these sera was ~10-fold lower when assessed with dOAc-1925wzzB-COPS and ~1,000-fold lower for SE COPS, which shares *Salmonella* O-antigens 1 and 12 and the core polysaccharide, indicating that the anti-COPS IgG immune response was largely STm-specific with a bias towards O-acetylated epitopes. Mice immunized with dOAc-STm-COPS^KDO^:CRM_197_ demonstrated a broader range of serum IgG titers against 1925wzzB-COPS with a GMT ~10-fold lower than that achieved with the O-acetylated STm-COPS^KDO^:CRM_197_ conjugate. The IgG GMT of the dOAc-STm-COPS^KDO^:CRM_197_ sera was comparable for both native and dOAc-1925wzzB-COPS. A greater proportion of these sera demonstrated antibody titers against SE COPS than was found for sera from mice immunized with STm-COPS^KDO^:CRM_197_. The proportional level of epitope-specific IgG in the sera of individual mice was generally consistent with the trend seen for the overall group GMT; however, some exceptions were evident (S6 Fig).

To assess the efficacy of these two vaccine preparations, conjugate-immunized mice and PBS controls were challenged in equal proportions with 1x10^6^ or 5x10^6^ CFU of STm D65, resulting in >95% mortality in PBS controls; mice receiving the higher dose succumbed more rapidly (Fig 5B). Mice immunized with the STm-COPS^KDO^:CRM_197_ conjugate were 100% protected against fatal infection with either challenge dose. Mice immunized with dOAc-STm-COPS^KDO^:CRM_197_ were significantly protected against challenge but at a lower level than mice immunized with STm-COPS^KDO^:CRM_197_ (63–74% VE versus 100% VE, *P* < 0.05).

The basis for the differential protective efficacy achieved by the CRM_197_ conjugates synthesized with native- and dOAc-1925wzzB-COPS was unclear since differences were found in both total anti-COPS IgG GMTs and O-epitope specificity patterns. Thus, analyses were undertaken to distinguish whether a qualitative difference (e.g., specificity, avidity) in the anti-COPS immune response or rather a quantitative difference in the anti-COPS IgG titer accounted for the higher protection achieved with the STm-COPS^KDO^:CRM_197_ conjugate. Serum bactericidal antibody (SBA) and opsonophagocytic antibody (OPA) assays were performed using equivalent anti-1925wzzB-COPS IgG ELISA units (EU). For these analyses, we selected sera that demonstrated different O-antigen specificity profiles (Fig 5C). Sera that contained antibodies predominantly against O-acetyl epitopes were identified by high reactivity with 1925wzzB-COPS but low reactivity with dOAc-1925wzzB-COPS. Conversely, sera manifesting antibody titers that were equivalent for native- and dOAc-1925wzzB-COPS but negligible for SE COPS were presumed to be directed primarily against O4. Remarkably, bactericidal activity among the different sera was indistinguishable when the anti-COPS IgG EU were equivalent (Fig 5D and 5E). Additionally, comparison of the anti-1925wzzB-COPS IgG titers induced by the two COPS^KDO^:CRM_197_ vaccines for mice that survived infection at the more restrictive higher challenge dose versus those that succumbed to infection, indicated that protection against challenge correlated with higher anti-polysaccharide antibody levels (S7 Fig).

### *In-silico* conformational analysis of STm OPS molecules

**Fig 6:**
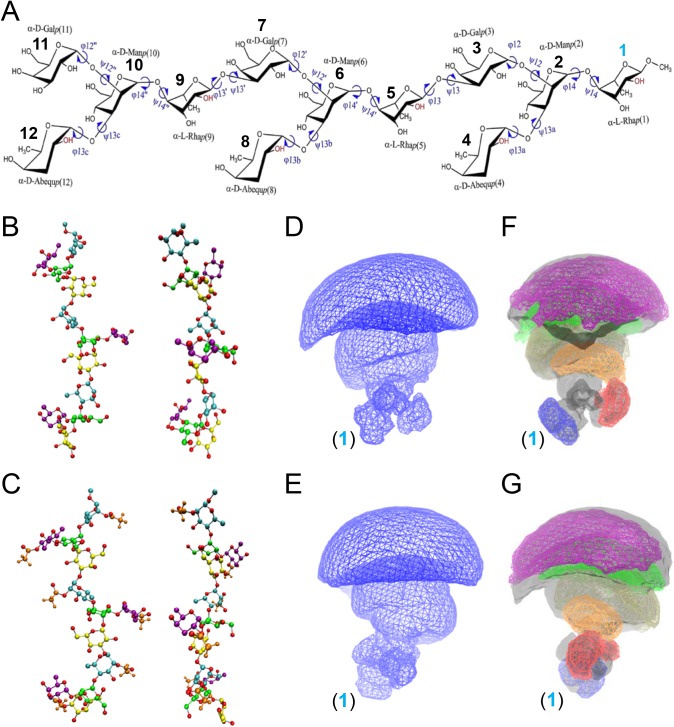
Chemical structure of the OPS used for computational simulations and chemical models and sampled volumes of the most prevalent conformational cluster “11111111”. (A) Structure of the base (O:4,12) 3-repeat STm polysaccharide unit used for computational analyses. Individual monosaccharide units are indicated numerically, with the reducing end rhamnose (Rha) designated as (1, blue). The representative O-acetylated polysaccharide was constructed with the hydroxyl group substituted by an acetyl at the C2 position of both α-D-Abequ*p* and α-L-Rha*p* (-OH, red). (B,C) Structural models of GL cluster “11111111” displaying 90° of rotation around the carbohydrate stem for the base (B) and O-acetylated base (C) polysaccharides. The acetyl group is shown in brown, glucose in blue, mannose in green, galactose in yellow, abequose in purple, and rhamnose in cyan. For clarity, all hydrogen atoms are omitted except those on the acetyl group. (D,E,F,G) 3D spatial distributions (wire frame) displaying 90° of rotation around the carbohydrate stem for the base (D,E) and O-acetylated base (F,G) polysaccharides for all non-hydrogen atoms. The reducing end Rha (1, blue), indicated at the base, is approximated as linked to the core polysaccharide or preceding OPS repeat. Conformational flexibility increases progressively relative to the reducing end anchor point. The base polysaccharide backbone is identified by a transparent grey surface overlaid with the individual O-acetyl groups in color for monosaccharides (1) [blue], (4) [red], (5) [orange], (8) [tan], (9) [green] and (12) [purple]. Contour levels are set to 10^−6^ for the full polysaccharide and to 10^−5^ for the individual acetyl groups.

Due to their multiple glycosidic linkages, polysaccharides are flexible and have the potential to assume different conformations. The conformational properties of STm OPS could possibly become altered upon acetylation, producing epitopes unique to O-acetylated polysaccharide repeats, and thereby influencing the pattern of antibody induction. To address this possibility, we conducted *in silico*-enhanced sampling molecular dynamics (MD) simulations to determine the accessible conformations of STm OPS and their relative frequencies upon O-acetylation. For these analyses, we modeled a base O:4,12 3-repeat molecule (Fig 6A) as well as variants that were either O-acetylated on rhamnose and abequose, glucosylated at the central or terminal repeat unit, or a combination thereof. Although slight differences were evident, we found that the all of these STm polysaccharides maintained the same dominant conformation (“11111111”) for at least 90% of the *in silico* simulations (Table 3). The modeled polysaccharides also demonstrated comparable patterns of total occupied 3D space based on the cumulative sum and high degree of overlap between the conformational volumes for the individual monosaccharide residues in the polysaccharide chain (S5 and S6 Tables). Representative native and O-acetylated saccharides in the dominant conformation “11111111” (Fig 6B and 6C) underwent 3D rendering, which displays the total volume occupied by each of these polysaccharide structures (Fig 6D and 6E). Examination of the abequose and adjacent OPS repeat rhamnose O-acetyls in the “11111111” conformation indicates that they are highly solvent accessible (S7 Table) and are in close spatial proximity (Fig 6F and 6G).

**Table 3:** Percent of total simulation time occupied by the accessible conformations of each STm OPS^a^.

GL Cluster Index^b^	3-repeat base PS^c^	*OAc* base PS^d^	*Glu* PS 1^e^	*Glu* PS 2^f^	*OAc + Glu* PS 1^d^^,^^e^	*OAc + Glu* PS 2^d^^,^^f^
11111111	91.1	91.0	96.6	94.3	96.2	90.8
11111112	0.0	0.6	0.2	0.1	0.0	0.7
11111121	0.0	0.4	0.1	0.1	0.4	0.0
11111211	0.0	1.4	0.1	0.4	0.1	1.6
11121111	0.0	0.0	0.3	0.2	0.1	0.0
11211111	2.2	1.1	0.0	1.4	0.2	1.8
12111111	4.4	1.6	0.8	1.5	0.3	0.6
12111112	0.0	0.0	0.0	0.0	0.0	0.0
12111211	0.0	0.0	0.0	0.0	0.0	0.0
12121111	0.0	0.0	0.0	0.0	0.0	0.0
12211111	0.0	0.0	0.0	0.0	0.0	0.0
21111111	1.7	3.7	1.9	2.1	2.6	4.3
21121111	0.0	0.2	0.0	0.0	0.0	0.0
21211111	0.2	0.0	0.1	0.0	0.0	0.1
22111111	0.1	0.0	0.0	0.0	0.1	0.0

^a^ % residence in the indicated conformation, only the clusters with a probability > 0.1% are listed.

^b^ Cluster index indicates orientation of each of the 8 glycosidic linkages in the different conformations.

^c^ O epitopes 4,12.

^d^ O-acetylation on Abe C2, Rha C2 for all 3 tetrasaccharide repeats.

^e^ Glucosylation on central repeat unit Gal.

^f^ Glucosylation on terminal (non-reducing end) repeat Gal.

## Discussion

The native COPS purified from our STm reagent strain was incompletely O-acetylated at abequose and rhamnose. Analyses of the OPS from Malawian STm strain D23580 and STm clinical isolates from Kenya found they were also incompletely O-acetylated at both abequose and rhamnose [11, 38]. Variability in OPS O-acetylation patterns among STm clinical isolates may have implications, however, for selection of a representative OPS hapten. Antibodies against O-acetyls may not bind efficiently to the underlying monosaccharide structure and binding by OPS backbone-specific antibodies may be reduced in the context of O-acetylation [41]. The close spatial proximity of the abequose and rhamnose O-acetyls also raises the possibility of unique epitopes formed by their combination. Sun-type glycoconjugates with 1925wzzB-COPS induced antibodies against both O-acetyl and OPS backbone epitopes and imparted high levels of protection against fatal challenge in mice.

O-acetyls are important immune determinants for several bacterial polysaccharide vaccines including *S*. Paratyphi A COPS-based glycoconjugates wherein the rhamnose O-acetyls in the trisaccharide backbone were found to be essential for induction of bactericidal antibodies [10, 42–44]. However, their importance to STm immunity was heretofore unclear. Protection by passive transfer of anti-O4 IgG mAbs in mice has been reported for both an O:4,5,12 STm as well as strain D23580 that is variably O-acetylated on both rhamnose and abequose [13, 45]. The functional activity of anti-O5 IgG mAbs is not well established; however, a monoclonal anti-O5 IgA protected mice against oral but not intraperitoneal STm challenge [13, 46]. We found that the functional bactericidal activity of anti-O-acetyl antibodies may not differ greatly from antibodies recognizing other STm OPS epitopes. This notion is in agreement with a recent report that found D23580 COPS conjugated to CRM_197_ induced antibodies that bound the homologous OPS more efficiently than COPS lacking O-acetyls or acetylated only at abequose, but maintained bactericidal activity against STm isolates that expressed all these OPS forms [47]. Protection may thus depend upon reaching a critical titer of antibody that is sufficient to bind the infecting isolate OPS, irrespective of epitope specificity. This is supported by our observation that higher anti-OPS titers correlated with survival in mice immunized with the CRM_197_-based COPS conjugates, wherein the COPS molecule was the sole protective antigen and the two vaccine constructs differed only in the presence or absence of polysaccharide O-acetyls. It is therefore reasonable that a partially O-acetylated polysaccharide would constitute the preferred polysaccharide vaccine antigen, as it could induce immunity against a wide range of structures, and thereby ensure broad coverage.

We also found that conjugate architecture contributed to immunogenicity. The lattice-type conjugate of 1925wzzB-COPS and FliC synthesized with an ADH linker (STm-COPS^Lat^:FliC) was less immunogenic and imparted lower protection than the sun-type conjugate generated with thiol-aminooxy and GMBS linkers (STm-COPS^KDO^:FliC). Although the lattice-type conjugate suffered from loss of OPS O-acetyls, the sun-type dOAc-STm-COPS^KDO^:CRM_197_, which similarly lacked O-acetyls, was immunogenic and protective. Differences in protein carrier function can influence the immunogenicity of glycoconjugates [48]; however, this was likely not a contributing factor here as sun-type conjugates of 1925wzzB-COPS (either FliC or CRM_197_) induced equally robust anti-COPS IgG levels. It is conceivable that the ADH linker may have exerted epitopic dominance, as can occur when the hapten is small and the linker highly immunogenic [49, 50]. The superior immunogenicity of conjugates generated by linkage through the polysaccharide reducing end is more likely accounted for, however, by minimal perturbation of OPS epitopes and the formation of a structure better able to cross-link B-cell receptors.

Our finding that STm OPS O-acetyls are immunodominant epitopes is in agreement with a prior report which found that mice immunized with an O:1,4,5,12 STm isolate displayed higher anti-LPS antibody titers compared with an isogenic derivative lacking abequose O-acetylation [51]. We also found that there was marked heterogeneity in the humoral response to conjugates generated with de-O-acetylated COPS compared to immunization with conjugates generated with the native O-acetylated polysaccharide. As other antigenic determinants were not altered upon de-O-acetylation, it is presumed that the disparity is due to loss of the O-acetyl groups. The high variance in anti-COPS antibody titers for the de-O-acetylated COPS conjugates is similar to our prior findings in mice immunized with SE COPS and FliC conjugates [18]. It was suggested that the differential immunogenicity of O-acetylated STm OPS may be accounted for by unique conformational epitopes [41, 51]. The *in silico* analyses conducted herein assessed preferred polysaccharide conformations defined by changes in the orientation around the glycosidic linkages [32]. This method also models the total 3D space occupied by all polysaccharide conformations, thus approximating the relative antigenic shape seen by the immune system. Our analyses suggest no alteration of STm OPS conformational properties upon O-acetylation. They further indicate a single dominant conformation in which the O-acetyls extend outward from the polysaccharide backbone and are highly solvent exposed. It is conceivable that the physicochemical properties of O-acetyls (e.g., size, partial charge, hydrophobicity) may provide a putative stabilizing interaction between the polysaccharide and the B-cell receptor and thus account for their pronounced immunogenicity. Further structural analyses will be needed to confirm these findings and assess the biophysical interactions between polysaccharide O-acetyls and antibodies.

We have reported previously that antibodies directed against NTS flagellin proteins mediated functional bactericidal activity in-vitro, and that passive transfer of a monoclonal antibody specific for STm FliC could protect mice against fatal infection with D65 [19]. By using flagellin as the carrier for STm COPS, protective immunity could thus be provided by both hapten and carrier components. The immunologic memory induced by glycoconjugate vaccines includes T cell responses to the carrier protein. It is thus conceivable that the anti-polysaccharide immune response in vaccinated individuals may thus be additionally boosted after natural *S*. Typhimurium infection due to T helper memory responses against flagellin peptides.

Development of a vaccine to control iNTS infections in sub-Saharan Africa remains an important global health priority. Further studies will also need to address a bivalent vaccine formulation of the sun-type STm COPS^KDO^:FliC with a comparably optimized SE COPS:FliC glycoconjugate. We elected to use FliC as the carrier protein to ensure coverage against serovar 1,4,[5],12:i:- that lacks phase 2 flagella expression [3]. It will also be important to determine whether cross-protection can be achieved against other sub-Saharan African group B serovars (e.g., *S*. Stanleyville) that have the same O type, but different H type, and constitute a low but nevertheless significant proportion of pediatric iNTS disease [3].

## Supporting information

S1 Fig Free energy landscapes of all glycosidic linkages (GL) derived from the native saccharide simulation. Results indicate the regions that are sampled during the simulations of the GLs as defined by the ϕ and Ψ dihedrals. The landscapes show that only one conformation of the 1→2 linkages are sampled during the simulations while two regions are sampled by the 1→3 and 1→4 linkages. For each GL, these two regions represent the 1 and 2 identifiers used for determination of the GL clusters.
(TIF)

S2 Fig Chemical structures of the α-L-rhamnose and α-D-abequose model compounds O-acetylated at the C2 position used in the parameterization of the acetyl group. (TIF)

S3 Fig Potential energy scans from the QM and MM levels of theory along the dihedral C3-C2-O2-CA2 in both acetylated α-D-abequose (top) and acetylated L-rhamnose (bottom) at the C2 position. The initial model was constructed with the directly transferred parameters and the final model was constructed with the fitted parameters.
(TIF)

S4 Fig Conjugate architecture and molecular size analysis of lattice and sun-type 1925wzzB-COPS conjugates with FliC. (A) Schematic of the conjugate architecture for lattice and sun-type conjugates. (B) HPLC-SEC chromatogram with A280 nm detection for STm-COPS^Lat^:FliC (long-dash line), STm-COPS^KDO^:FliC (solid line), and unconjugated FliC (short-dash line).
(TIF)

S5 Fig Reactivity of STm-COPS^KDO^:CRM_197_ conjugates with monoclonal antibodies against O4 and O5. ELISA reactivity of 1925wzzB-COPS (black circles), STm-COPS^KDO^:CRM_197_ (grey circles), and dOAc-STm-COPS^KDO^:CRM_197_ (open circles) with either an anti-O4 monoclonal antibody or an anti-O5 monoclonal antibody.
(TIF)

S6 Fig Proportional reactivity of individual sera induced by STm-COPS:CRM_197_ conjugates for different STm COPS epitopes. Antibody proportions for different STm COPS epitopes were determined for individual sera from mice immunized with STm-COPS^KDO^:CRM_197_ (A) or dOAc-STm-COPS^KDO^:CRM_197_ (B). Proportional epitope specific antibody levels (left vertical axis) are represented by vertical bars as indicated in the figure legend. Total anti-1925wzzB-COPS IgG titers (right vertical axis) are denoted for each serum sample (open circles). Sera from mice that succumbed to infection after challenge are indicated by asterisk.
(TIF)

S7 Fig Serum anti-1925wzzB-COPS IgG titers from COPS^KDO^:CRM_197_-immunized mice, stratified by survival status after challenge with STm D65. Serum IgG titers for 1925wzzB-COPS from mice immunized with STm-COPS^KDO^:CRM_197_ (grey circles, *n* = 20) or dOAc-STm-COPS^KDO^:CRM_197_ (open circles, *n* = 20) were grouped by survival status after challenge with 5x10^6^ CFU of STm D65. Solid bars indicate the GMT; comparisons between groups were accomplished by a two-tailed Mann-Whitney U test.
(TIF)

S1 Table List of *Salmonella* strains used in this study. (DOCX)

S2 Table Simulation systems and duration of the simulations. (DOCX)

S3 Table Partitioning of the free energy landscape along ϕ/Ψ for dihedral based clustering analysis. (DOCX)

S4 Table HPAEC-PAD monosaccharide analyses of OPS repeat glucosylation in depolymerized *Salmonella* COPS. (DOCX)

S5 Table 3D spatial volume (Å^3^) sampled by each monosaccharide unit in the studied polysaccharides. (DOCX)

S6 Table Similarity of the spatial distributions sampled by the saccharides indicated by the overlap coefficient (*OC*)^a^ of the total volumes sampled by the respective systems. (DOCX)

S7 Table Solvent accessible surface area (SASA) in Å^2^ of the acetyl group in different O-acetylated saccharides. (DOCX)
