# Supplementary figures and images for: Development of a glycoconjugate vaccine to prevent invasive Salmonella Typhimurium infections in sub-Saharan Africa
Source: PLoS Negl Trop Dis. 2017 Apr 7;11(4):e0005493. doi: 10.1371/journal.pntd.0005493 (PMC5397072; doi:10.1371/journal.pntd.0005493)

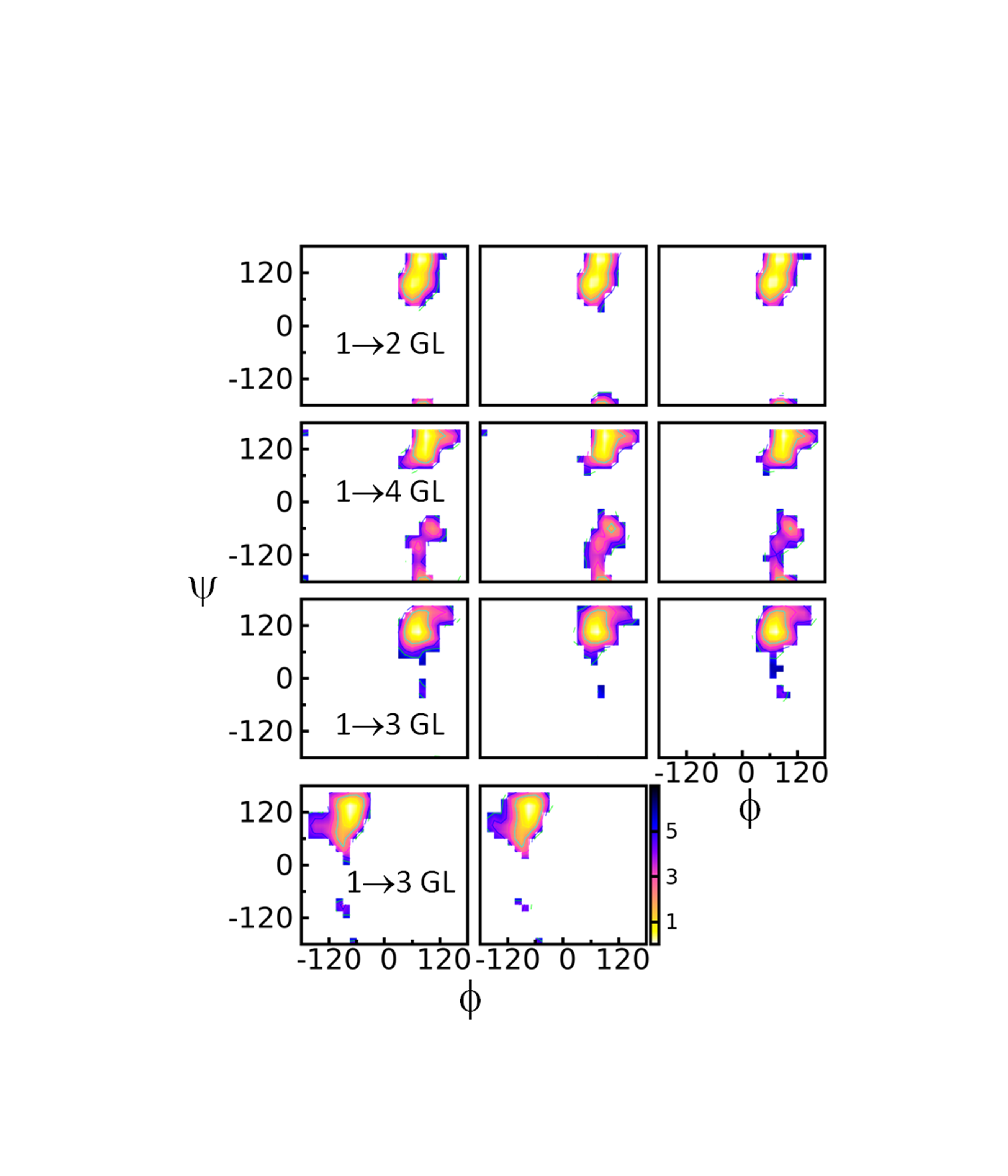

Supplement: S1 Fig — Results indicate the regions that are sampled during the simulations of the GLs as defined by the ϕ and Ψ dihedrals. The landscapes show that only one conformation of the 1→2 linkages are sampled during the simulations while two regions are sampled by the 1→3 and 1→4 linkages. For each GL, these two regions represent the 1 and 2 identifiers used for determination of the GL clusters. (TIF) [file pntd.0005493.s001.tif]

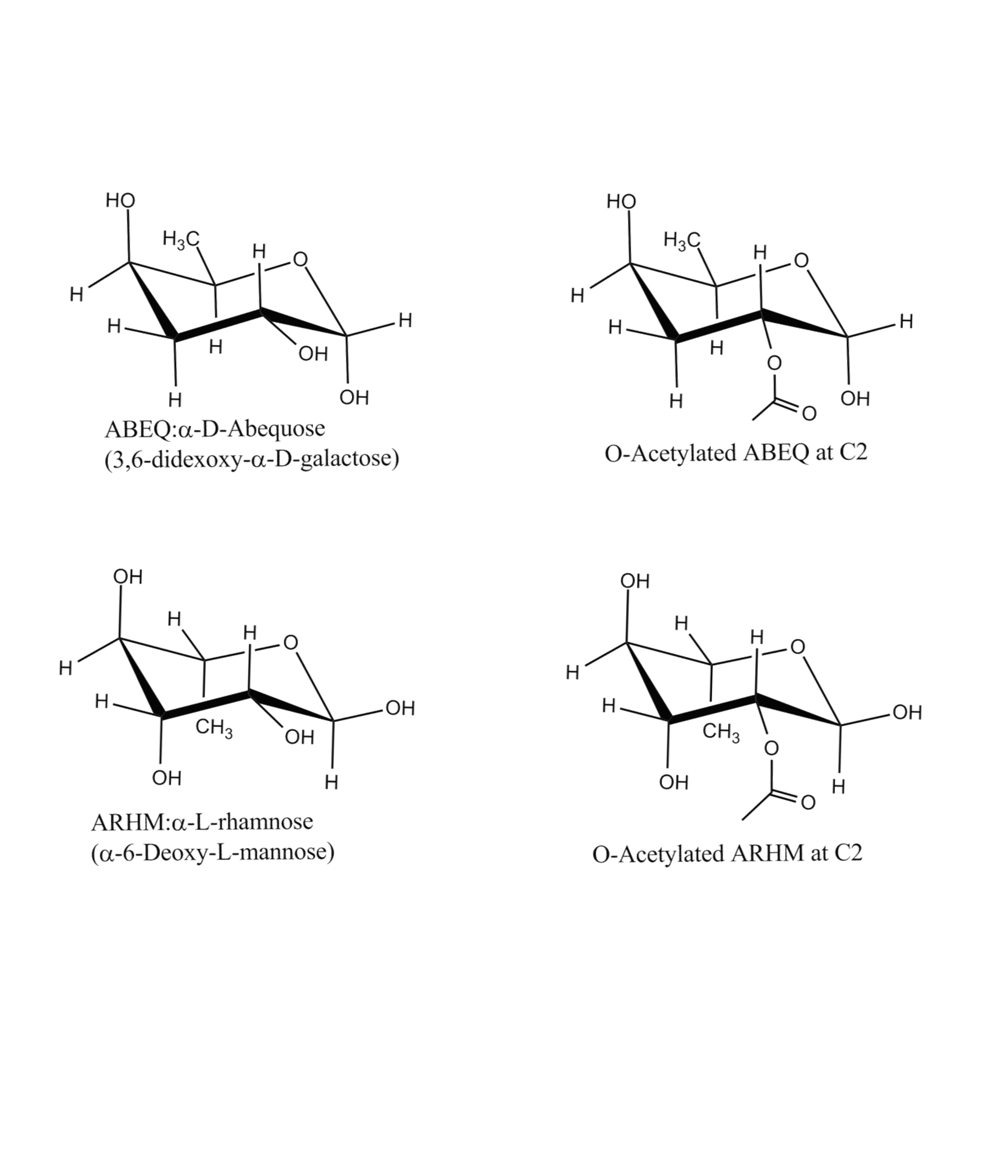

Supplement: S2 Fig — (TIF) [file pntd.0005493.s002.tif]

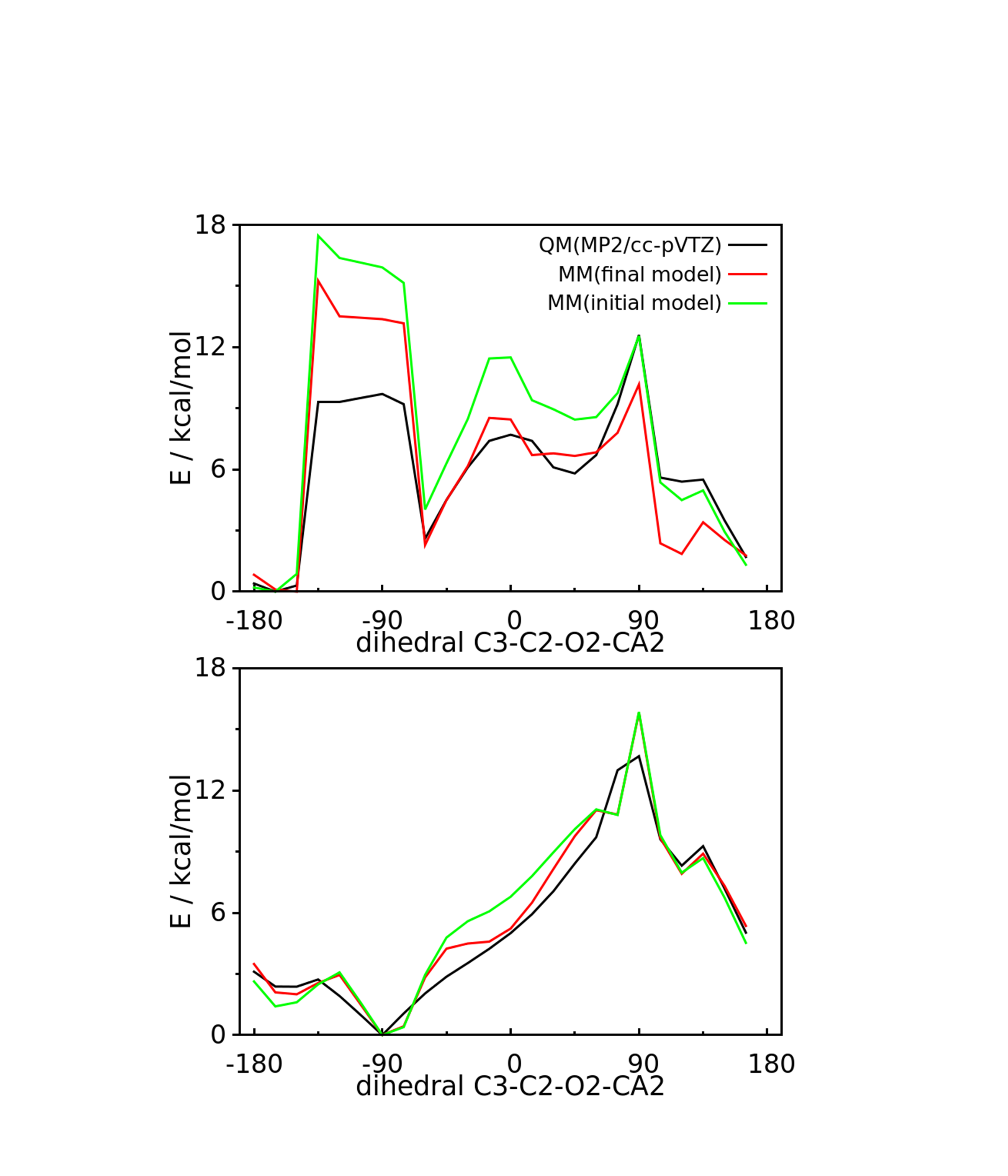

Supplement: S3 Fig — The initial model was constructed with the directly transferred parameters and the final model was constructed with the fitted parameters. (TIF) [file pntd.0005493.s003.tif]

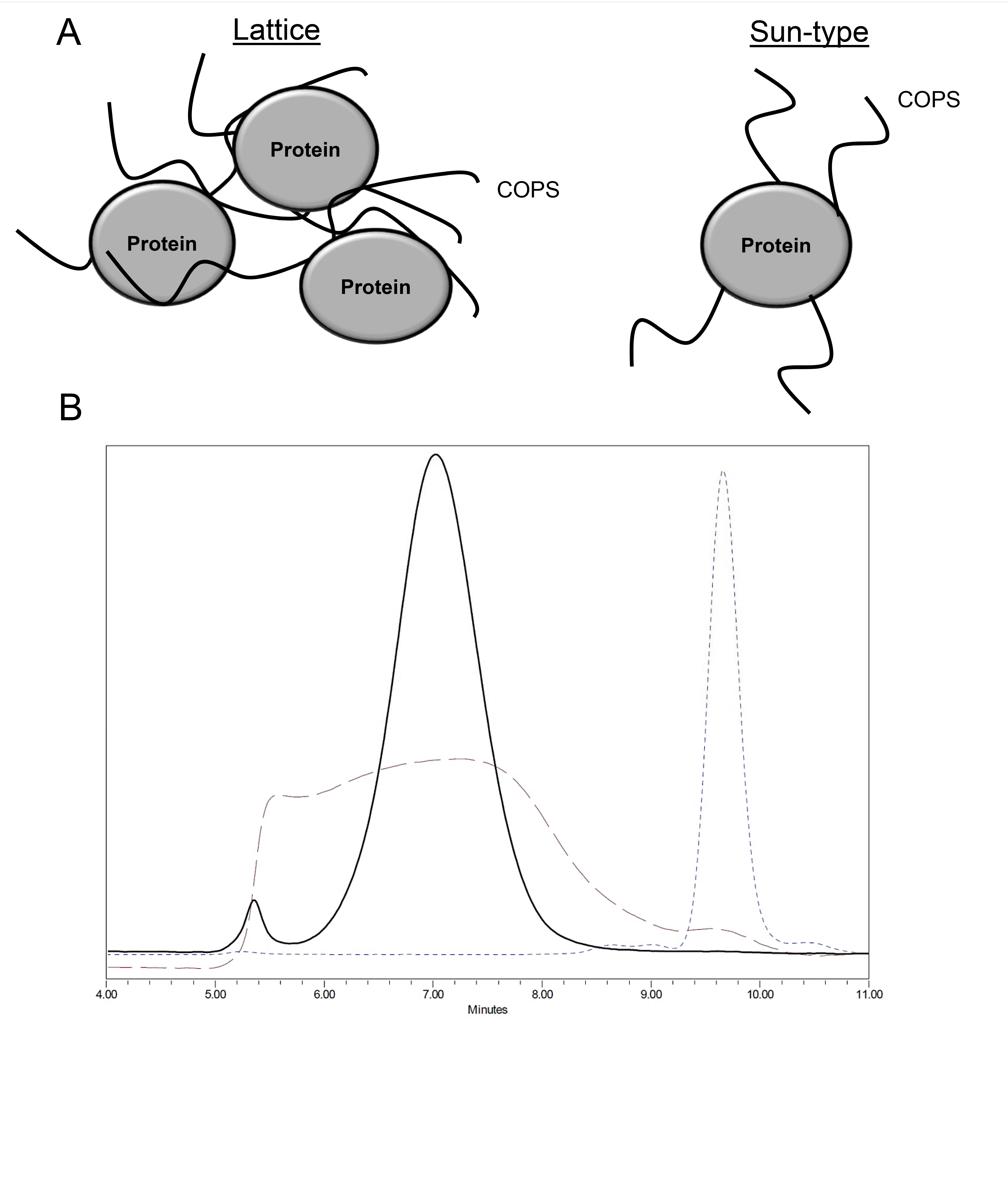

Supplement: S4 Fig — (A) Schematic of the conjugate architecture for lattice and sun-type conjugates. (B) HPLC-SEC chromatogram with A280 nm detection for STm-COPSLat:FliC (long-dash line), STm-COPSKDO:FliC (solid line), and unconjugated FliC (short-dash line). (TIF) [file pntd.0005493.s004.tif]

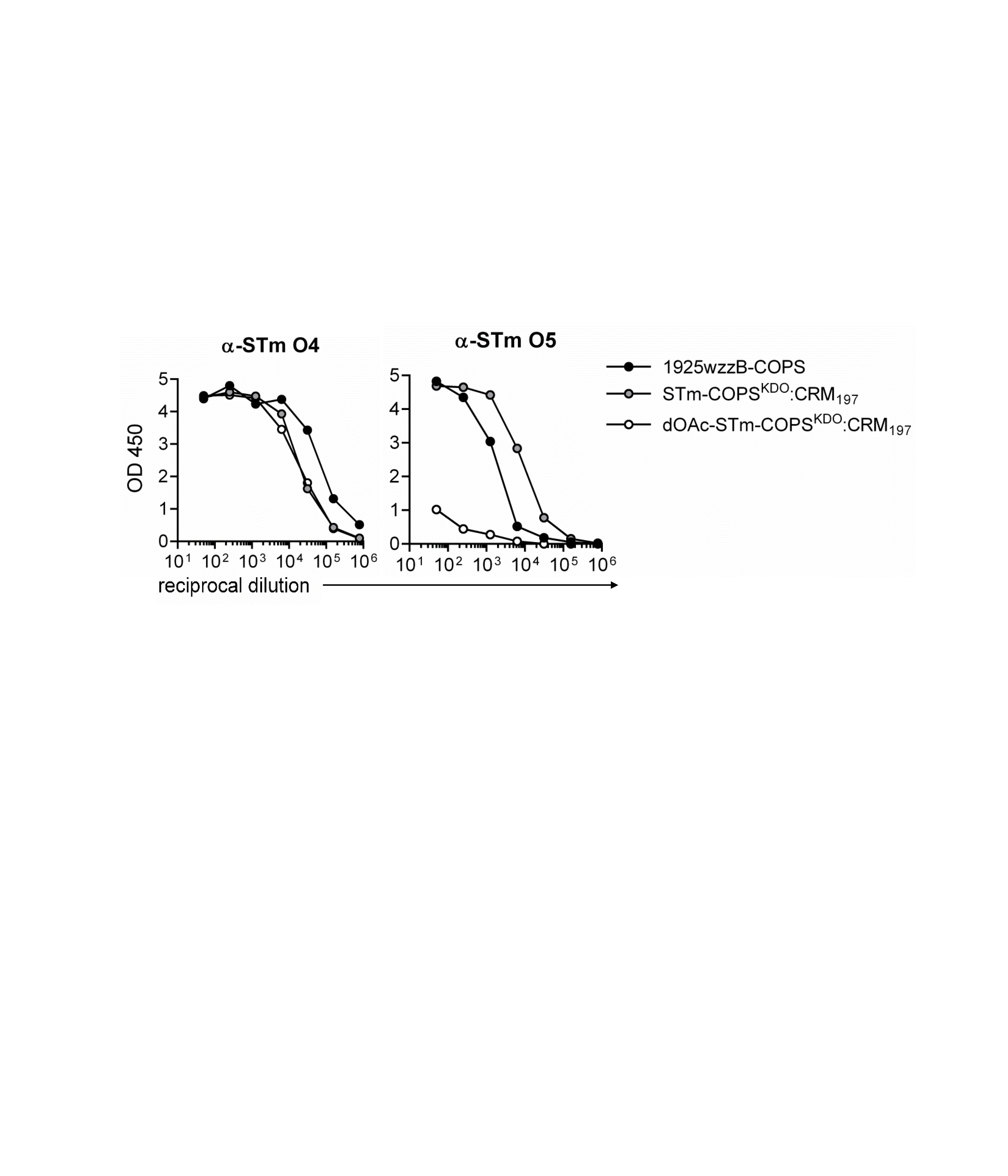

Supplement: S5 Fig — ELISA reactivity of 1925wzzB-COPS (black circles), STm-COPSKDO:CRM197 (grey circles), and dOAc-STm-COPSKDO:CRM197 (open circles) with either an anti-O4 monoclonal antibody or an anti-O5 monoclonal antibody. (TIF) [file pntd.0005493.s005.tif]

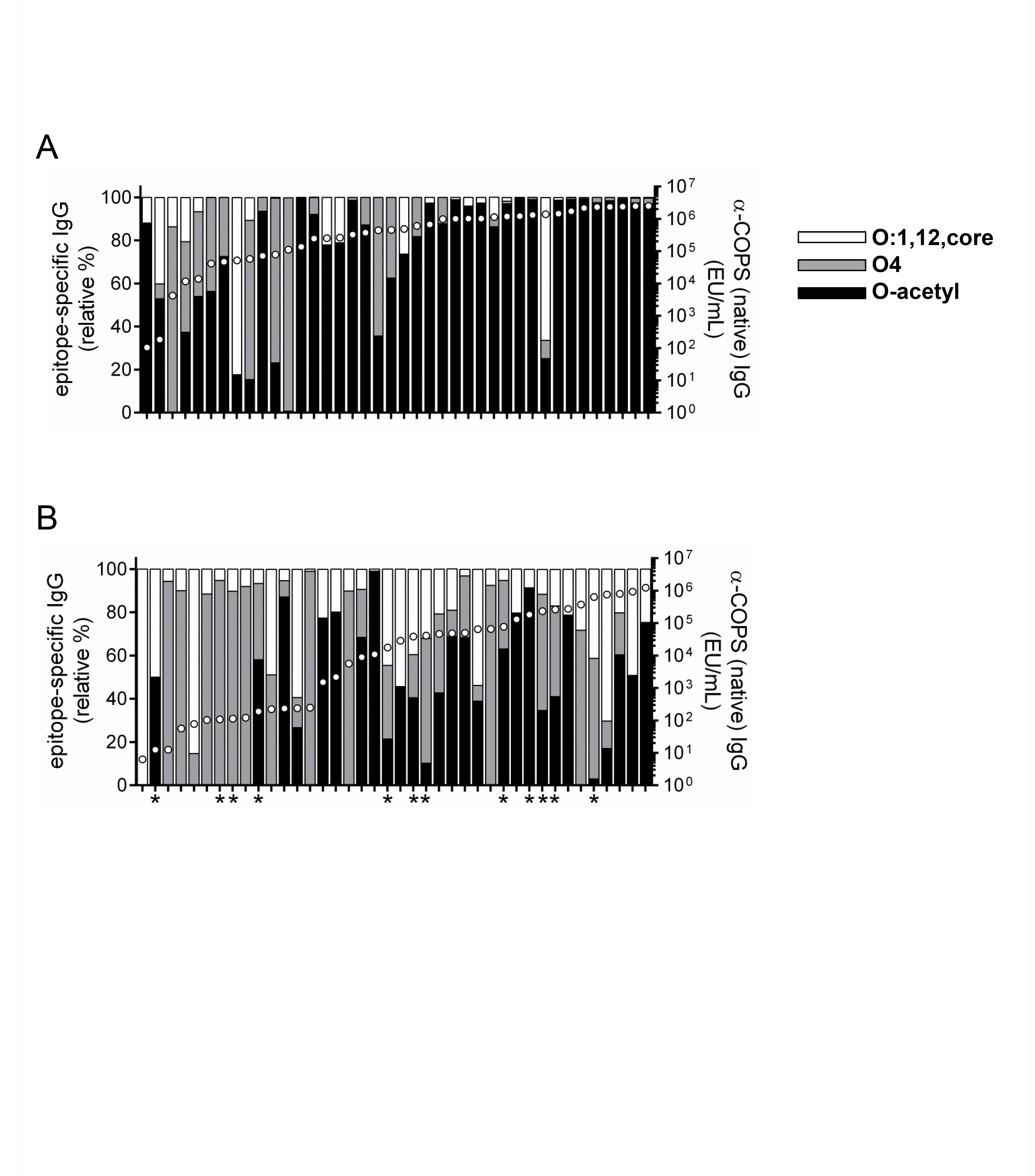

Supplement: S6 Fig — Antibody proportions for different STm COPS epitopes were determined for individual sera from mice immunized with STm-COPSKDO:CRM197 (A) or dOAc-STm-COPSKDO:CRM197 (B). Proportional epitope specific antibody levels (left vertical axis) are represented by vertical bars as indicated in the figure legend. Total anti-1925wzzB-COPS IgG titers (right vertical axis) are denoted for each serum sample (open circles). Sera from mice that succumbed to infection after challenge are indicated by asterisk. (TIF) [file pntd.0005493.s006.tif]

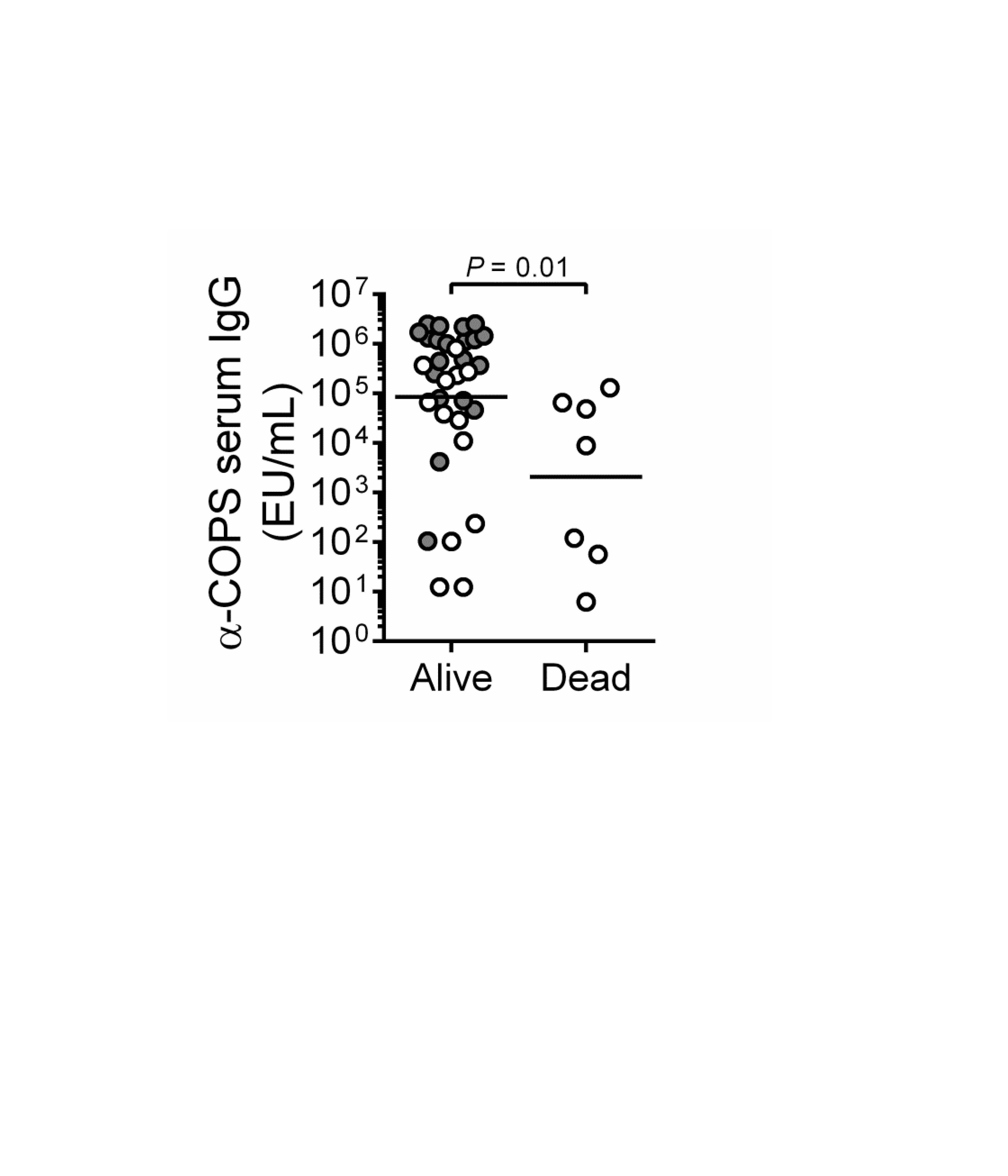

Supplement: S7 Fig — Serum IgG titers for 1925wzzB-COPS from mice immunized with STm-COPSKDO:CRM197 (grey circles, n = 20) or dOAc-STm-COPSKDO:CRM197 (open circles, n = 20) were grouped by survival status after challenge with 5x10^6^ CFU of STm D65. Solid bars indicate the GMT; comparisons between groups were accomplished by a two-tailed Mann-Whitney U test. (TIF) [file pntd.0005493.s007.tif]
