# Supplementary material for: Development of a glycoconjugate vaccine to prevent invasive Salmonella Typhimurium infections in sub-Saharan Africa
Source: PLoS Negl Trop Dis. 2017 Apr 7;11(4):e0005493. doi: 10.1371/journal.pntd.0005493 (PMC5397072; doi:10.1371/journal.pntd.0005493)
Supplement: S1 Table — (DOCX) [file pntd.0005493.s008.docx]

| **Table S1.** List of *Salmonella* strains used in this study | | | |
| --- | --- | --- | --- |
| **Serovar** | **Strain** | **Source/characteristics** | **References** |
| *S.* Typhimurium | D65 | Invasive isolate, 2002, Mali | [1-3] |
|  | A13 | Invasive isolate, 2002, Mali |  |
|  | P142 | Invasive isolate, 2006, Mali |  |
|  | Q65 | Invasive isolate, 2007, Mali |  |
|  | S42 | Invasive isolate, 2008, Mali |  |
|  | D23580 | Invasive isolate, Malawi | [4] |
|  | CVD 1931 | *S*. Typhimurium D65 *ΔguaBA ΔclpX* | [5] |
|  | CVD 1925 | *S*. Typhimurium I77 *ΔguaBA ΔclpP ΔfljB ΔfliD* | [6] |
|  | CVD 1925 (pSEC10-*wzzB*) | *S*. Typhimurium I77 *ΔguaBA ΔclpP ΔfljB ΔfliD* carrying plasmid pSEC10-*wzzB* | Hegerle, N. and Tennant, S.M. (manuscript in preparation) |
| *S.* Enteritidis | R11 | Clinical isolate, 2007, Mali | [1-3] |
|  | CVD 1943 | *S*. Enteritidis R11 *ΔguaBA ΔclpP* | [6] |

**References.**

1. Levy H, Diallo S, Tennant SM, Livio S, Sow SO, Tapia M, et al. PCR method to identify *Salmonella enterica* serovars Typhi, Paratyphi A, and Paratyphi B among *Salmonella* Isolates from the blood of patients with clinical enteric fever. J Clin Microbiol. 2008;46(5):1861-6. doi: 10.1128/JCM.00109-08. PubMed PMID: 18367574; PubMed Central PMCID: PMCPMC2395068.

2. Tennant SM, Diallo S, Levy H, Livio S, Sow SO, Tapia M, et al. Identification by PCR of non-typhoidal *Salmonella enterica* serovars associated with invasive infections among febrile patients in Mali. PLoS Negl Trop Dis. 2010;4(3):e621. doi: 10.1371/journal.pntd.0000621. PubMed PMID: 20231882; PubMed Central PMCID: PMCPMC2834738.

3. Tapia MD, Tennant SM, Bornstein K, Onwuchekwa U, Tamboura B, Maiga A, et al. Invasive Nontyphoidal *Salmonella* Infections Among Children in Mali, 2002-2014: Microbiological and Epidemiologic Features Guide Vaccine Development. Clin Infect Dis. 2015;61 Suppl 4:S332-8. doi: 10.1093/cid/civ729. PubMed PMID: 26449949; PubMed Central PMCID: PMCPMC4596934.

4. MacLennan CA, Gondwe EN, Msefula CL, Kingsley RA, Thomson NR, White SA, et al. The neglected role of antibody in protection against bacteremia caused by nontyphoidal strains of *Salmonella* in African children. J Clin Invest. 2008;118(4):1553-62. Epub 2008/03/22. doi: 10.1172/JCI33998. PubMed PMID: 18357343; PubMed Central PMCID: PMC2268878.

5. Tennant SM, Schmidlein P, Simon R, Pasetti MF, Galen JE, Levine MM. Refined Live Attenuated *Salmonella enterica* Serovar Typhimurium and Enteritidis Vaccines Mediate Homologous and Heterologous Serogroup Protection in Mice. Infect Immun. 2015;83(12):4504-12. doi: 10.1128/IAI.00924-15. PubMed PMID: 26351285; PubMed Central PMCID: PMCPMC4645371.

6. Tennant SM, Wang JY, Galen JE, Simon R, Pasetti MF, Gat O, et al. Engineering and preclinical evaluation of attenuated nontyphoidal *Salmonella* strains serving as live oral vaccines and as reagent strains. Infect Immun. 2011;79(10):4175-85. doi: 10.1128/IAI.05278-11. PubMed PMID: 21807911; PubMed Central PMCID: PMC3187273.
