# Supplementary material for: Development of a glycoconjugate vaccine to prevent invasive Salmonella Typhimurium infections in sub-Saharan Africa
Source: PLoS Negl Trop Dis. 2017 Apr 7;11(4):e0005493. doi: 10.1371/journal.pntd.0005493 (PMC5397072; doi:10.1371/journal.pntd.0005493)
Supplement: S2 Table — (DOCX) [file pntd.0005493.s009.docx]

| **Table S2.** Simulation systems and duration of the simulations | | |
| --- | --- | --- |
| **Models**^a^ | **simulation duration/ns^d^** | **Program** |
| 3-repeat base tetrasaccharide | 100 | CHARMM |
| O-acetylated base PS | 100 | CHARMM-OPENMM |
| glucosylated PS 1^b^ | 60 | CHARMM |
| glucosylated PS 2^c^ | 60 | CHARMM |
| O-acetylated glucosylated PS 1^a,b^ | 60 | CHARMM |
| O-acetylated glucosylated PS 2 ^a,c^ | 60 | CHARMM |
| ^a^ Designations described in Table 3 | | |
