# Supplementary material for: Development of a glycoconjugate vaccine to prevent invasive Salmonella Typhimurium infections in sub-Saharan Africa
Source: PLoS Negl Trop Dis. 2017 Apr 7;11(4):e0005493. doi: 10.1371/journal.pntd.0005493 (PMC5397072; doi:10.1371/journal.pntd.0005493)
Supplement: S3 Table — (DOCX) [file pntd.0005493.s010.docx]

| **Table S3.** Partitioning of the free energy landscape along φ/ψ for dihedral based clustering analysis | | | | |
| --- | --- | --- | --- | --- |
| Glycosidic linkage dihedrals | 1 | 2 | 3 | 4 |
| ϕ14/ψ14 | (-180°, -120°) & (0°, 180°]/(-180°, -120°) & (0°, 180°] | (-180°, -120°) & (0°, 180°] /[-120°, 0°] | [-120°, 0°]/(-180°, -120°) & (0°, 180°] | [-120°, 0°]/[-120°, 0°] |
| ϕ14′/ψ14′ | (-180°, -120°) & (0°, 180°]/(-180°, -120°) & (0°, 180°] | (-180°, -120°) & (0°, 180°] /[-120°, 0°] | [-120°, 0°]/(-180°, -120°) & (0°, 180°] | [-120°, 0°]/[-120°, 0°] |
| ϕ14′′/ψ14′′ | (-180°, -120°) & (0°, 180°]/(-180°, -120°) & (0°, 180°] | (-180°, -120°) & (0°, 180°] /[-120°, 0°] | [-120°, 0°]/(-180°, -120°) & (0°, 180°] | [-120°, 0°]/[-120°, 0°] |
| ϕ13/ψ13 | (-180°, 0°) & (120°, 180°]/(-180°, -120°) & (0°, 180°] | (-180°, 0°) & (120°, 180°]/[-120°, 0°] | [0°,120°]/°]/(-180°, -120°) & (0°, 180°] | [0°,120°]/[-120°, 0°] |
| ϕ13′/ψ13′ | (-180°, 0°) & (120°, 180°]/(-180°, -120°) & (0°, 180°] | (-180°, 0°) & (120°, 180°]/[-120°, 0°] | [0°,120°]/°]/(-180°, -120°) & (0°, 180°] | [0°,120°]/[-120°, 0°] |
| ϕ13a/ψ13a | (-180°, 0°) & (120°, 180°]/(-180°, -120°) & (0°, 180°] | (-180°, 0°) & (120°, 180°]/[-120°, 0°] | [0°,120°]/°]/(-180°, -120°) & (0°, 180°] | [0°,120°]/[-120°, 0°] |
| ϕ13b/ψ13b | (-180°, 0°) & (120°, 180°]/(-180°, -120°) & (0°, 180°] | (-180°, 0°) & (120°, 180°]/[-120°, 0°] | [0°,120°]/°]/(-180°, -120°) & (0°, 180°] | [0°,120°]/[-120°, 0°] |
| ϕ13c/ψ13c | (-180°, 0°) & (120°, 180°]/(-180°, -120°) & (0°, 180°] | (-180°, 0°) & (120°, 180°]/[-120°, 0°] | [0°,120°]/°]/(-180°, -120°) & (0°, 180°] | [0°,120°]/[-120°, 0°] |
