# Supplementary material for: Development of a glycoconjugate vaccine to prevent invasive Salmonella Typhimurium infections in sub-Saharan Africa
Source: PLoS Negl Trop Dis. 2017 Apr 7;11(4):e0005493. doi: 10.1371/journal.pntd.0005493 (PMC5397072; doi:10.1371/journal.pntd.0005493)
Supplement: S4 Table — (DOCX) [file pntd.0005493.s011.docx]

| **Table S4.** HPAEC-PAD monosaccharide analyses of OPS repeat glucosylation in depolymerized *Salmonella* COPS | |
| --- | --- |
| **COPS source** | **Glucose to rhamnose ratio** |
| CVD 1925 (pSEC10-*wzzB*) COPS | 0.12 |
| CVD 1943 COPS | 0.25 |
| D65 COPS | 0.19 |
