# Supplementary material for: Development of a glycoconjugate vaccine to prevent invasive Salmonella Typhimurium infections in sub-Saharan Africa
Source: PLoS Negl Trop Dis. 2017 Apr 7;11(4):e0005493. doi: 10.1371/journal.pntd.0005493 (PMC5397072; doi:10.1371/journal.pntd.0005493)
Supplement: S5 Table — (DOCX) [file pntd.0005493.s012.docx]

| **Table S5.** 3D spatial volume (Å^3^) sampled by each monosaccharide unit in the studied polysaccharides^a^ | | | | | | |
| --- | --- | --- | --- | --- | --- | --- |
| **monosaccharide index (position)^b^** | **3-repeat base tetrasaccharide** | **O-acetylated base PS** | **glucosylated PS 1** | **glucosylated PS 2** | **O-acetylated glucosylated PS 1** | **O-acetylated glucosylated PS 2** |
| 1 | 63 | 61 | 58 | 62 | 60 | 63 |
| 2 | 49 | 47 | 39 | 45 | 47 | 45 |
| 3 | 65 | 65 | 65 | 63 | 66 | 64 |
| 4 | 71 | 76 | 69 | 71 | 81 | 77 |
| 5 | 360 | 354 | 332 | 335 | 327 | 356 |
| 6 | 1268 | 1182 | 1102 | 1136 | 1013 | 1167 |
| 7 | 3731 | 3521 | 3211 | 3428 | 2936 | 3104 |
| 8 | 3558 | 3386 | 3146 | 3226 | 2989 | 3158 |
| 9 | 7483 | 6092 | 5942 | 6064 | 5162 | 5495 |
| 10 | 13619 | 10999 | 10999 | 10638 | 9428 | 9358 |
| 11 | 24448 | 19912 | 18835 | 18607 | 16605 | 16688 |
| 12 | 22747 | 19237 | 17996 | 18301 | 15755 | 16115 |
| Total (sum) | 77462 | 64932 | 61794 | 61976 | 54469 | 55690 |
| ^a^ Each conformation was first aligned with the ring atoms in the first tetrasaccharide unit and then the sampled volume was computed as the occupied number of the 3D grid map by the ring atoms of each monosaccharide. Polysaccharide designations described in Table 3.  ^b^ The monosaccharide index is defined in Figure 4A | | | | | | |
