# Supplementary material for: Development of a glycoconjugate vaccine to prevent invasive Salmonella Typhimurium infections in sub-Saharan Africa
Source: PLoS Negl Trop Dis. 2017 Apr 7;11(4):e0005493. doi: 10.1371/journal.pntd.0005493 (PMC5397072; doi:10.1371/journal.pntd.0005493)
Supplement: S6 Table — (DOCX) [file pntd.0005493.s013.docx]

| **Table S6.** Similarity of the spatial distributions sampled by the saccharides indicated by the overlap coefficient (*OC*)^a^ of the total volumes sampled by the respective systems | | | | | | |
| --- | --- | --- | --- | --- | --- | --- |
|  | **3-repeat base tetrasaccharide**^b^ | **O-acetylated base PS** | **glucosylated PS 1** | **glucosylated PS 2** | **O-acetylated glucosylated PS 1** | **O-acetylated glucosylated PS 2** |
| native saccharide | 1.00 (0.84) | 0.73 | 0.79 | 0.86 | 0.71 | 0.74 |
| O-acetylated saccharide | 0.73 | 1.00 (0.77) | 0.74 | 0.76 | 0.79 | 0.86 |
| glucosylated saccharide1 | 0.79 | 0.74 | 1.00 (0.80) | 0.82 | 0.80 | 0.73 |
| glucosylated saccharide2 | 0.86 | 0.76 | 0.82 | 1.00 (0.80) | 0.76 | 0.77 |
| glucosylated and O-acetylated saccharide1 | 0.71 | 0.79 | 0.80 | 0.76 | 1.00 (0.82) | 0.78 |
| glucosylated and O-acetylated saccharide2 | 0.74 | 0.86 | 0.73 | 0.77 | 0.78 | 1.00 (0.81) |
| ^a^ The overlap coefficient (*OC*) was computed with $OC=\sum_{i=1}^{N} \min\left(\frac{n_{i}^{A}}{\sum_{j=1}^{N} n_{j}^{A}},\frac{n_{i}^{B}}{\sum_{j=1}^{N} n_{j}^{B}}\right)$, where *N* is the number of voxels in the 3D grid map; $n_{i}^{A}$ and $n_{i}^{B}$ are the number of occupancy at the i-th voxel for saccharide molecules A and B, respectively. Each conformation was first aligned with the ring atoms in the first tetrasaccharide unit and then each ring atom in every conformation of a saccharide molecule was assigned to a specific voxel based on its Cartesian coordinate. The values in parenthesis are overlap coefficients computed from two halves of the total trajectory for each system.  ^b^ Designations described in Table 3 | | | | | | |
