# Supplementary material for: Development of a glycoconjugate vaccine to prevent invasive Salmonella Typhimurium infections in sub-Saharan Africa
Source: PLoS Negl Trop Dis. 2017 Apr 7;11(4):e0005493. doi: 10.1371/journal.pntd.0005493 (PMC5397072; doi:10.1371/journal.pntd.0005493)
Supplement: S7 Table — (DOCX) [file pntd.0005493.s014.docx]

| **Table S7.** Solvent accessible surface area (SASA) in Å^2^ of the acetyl group in different O-acetylated saccharides^a^ | | | | | | |
| --- | --- | --- | --- | --- | --- | --- |
| **monosaccharide index (position)^b^** | **O-acetylated base PS^d^** | | **O-acetylated glucosylated PS 1** | | **O-acetylated glucosylated PS 2** | |
| 1 | 70.9 (3.1) | 72.4 (2.5) | 71.2 (3.2) | 72.4 (2.5) | 71.2 (3.1) | 72.6 (2.5) |
| 4 | 69.6 (5.6) | 78.4 (2.5) | 70.1 (5.6) | 78.5 (2.5) | 70.0 (4.9) | 78.6 (2.6) |
| 5 | 62.7 (7.9) | 72.7 (2.3) | 62.1 (8.0) | 72.7 (2.4) | 62.8 (8.6) | 72.7 (2.4) |
| 8 | 70.0 (5.2) | 78.5 (2.5) | 70.2 (5.2) | 78.6 (2.5) | 70.6 (4.7) | 78.6 (2.5) |
| 9 | 63.1 (7.7) | 72.7 (2.4) | 61.1 (7.4) | 72.6 (2.4) | 63.5 (7.6) | 72.7 (2.3) |
| 12 | 72.0 (3.6) | 78.5 (2.5) | 71.6 (4.0) | 78.4 (2.4) | 71.9 (3.4) | 78.6 (2.4) |
| ^a^ A probe radius of 1.4 Å was used to compute the accessible solvent surface.(7) For each saccharide system, the value in the left column is the SASA in the full saccharide and the right column is the SASA in the respective monosaccharide that has been removed from the remainder of the saccharide systems, representing the maximum possible SASA of each acetyl group.  ^b^ The monosaccharide index is defined in Figure 4A  ^a^ O-acetylation on Abe C2, Rha C2 for all 3 tetrasaccharide repeats  ^b^ glucosylated at the central tetrasaccharide unit Gal  ^c^ glucosylated at the terminal tetrasaccharide unit Gal | | | | | | |
